# Creatine riboside is a cancer cell–derived metabolite associated with arginine auxotrophy

**DOI:** 10.1172/JCI157410

**Published:** 2022-07-15

**Authors:** Amelia L. Parker, Leila Toulabi, Takahiro Oike, Yasuyuki Kanke, Daxeshkumar Patel, Takeshi Tada, Sheryse Taylor, Jessica A. Beck, Elise Bowman, Michelle L. Reyzer, Donna Butcher, Skyler Kuhn, Gary T. Pauly, Kristopher W. Krausz, Frank J. Gonzalez, S. Perwez Hussain, Stefan Ambs, Bríd M. Ryan, Xin Wei Wang, Curtis C. Harris

**Affiliations:** 1 Laboratory of Human Carcinogenesis, Center for Cancer Research, National Cancer Institute (NCI), NIH, Bethesda, Maryland, USA.; 2 National Research Resource for Imaging Mass Spectrometry, Vanderbilt University, Nashville, Tennessee, USA.; 3 Pathology and Histotechnology Laboratory, Frederick National Laboratory, Frederick, Maryland, USA.; 4 Center for Cancer Research Collaborative Bioinformatics Resource,; 5 Chemical Biology Laboratory,; 6 Laboratory of Metabolism, and; 7 Liver Cancer Program, Center for Cancer Research, NCI, NIH, Bethesda, Maryland, USA.

**Keywords:** Oncology, Cancer, Lung cancer

## Abstract

The metabolic dependencies of cancer cells have substantial potential to be exploited to improve the diagnosis and treatment of cancer. Creatine riboside (CR) is identified as a urinary metabolite associated with risk and prognosis in lung and liver cancer. However, the source of high CR levels in patients with cancer as well as their implications for the treatment of these aggressive cancers remain unclear. By integrating multiomics data on lung and liver cancer, we have shown that CR is a cancer cell–derived metabolite. Global metabolomics and gene expression analysis of human tumors and matched liquid biopsies, together with functional studies, revealed that dysregulation of the mitochondrial urea cycle and a nucleotide imbalance were associated with high CR levels and indicators of a poor prognosis. This metabolic phenotype was associated with reduced immune infiltration and supported rapid cancer cell proliferation that drove aggressive tumor growth. CR^hi^ cancer cells were auxotrophic for arginine, revealing a metabolic vulnerability that may be exploited therapeutically. This highlights the potential of CR not only as a poor-prognosis biomarker but also as a companion biomarker to inform the administration of arginine-targeted therapies in precision medicine strategies to improve survival for patients with cancer.

## Graphical abstract

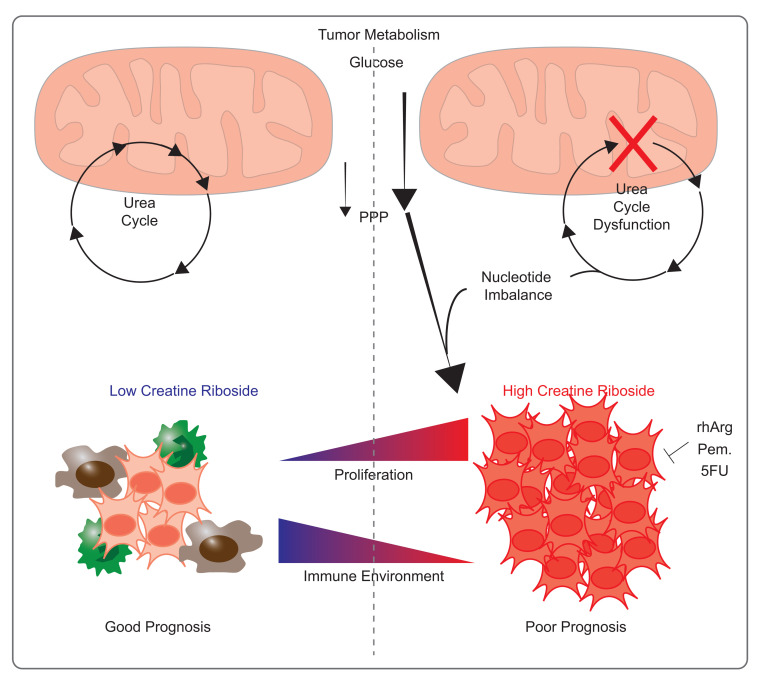

## Introduction

The reprogramming of cancer cell metabolism is increasingly appreciated for its central role in enabling the growth of the primary tumor as well as its metastasis to distant organs (1–3). These metabolic dependencies may be exploited not only to diagnose cancer but also to target cancer cells, while reducing side effects (3–5). Biomarkers predicting therapeutic efficacy have enabled the administration of targeted therapies to substantially improve the survival of a subset of patients with lung cancer and other poor-prognosis tumors in recent years (6, 7). This demonstrates the potential of metabolic biomarkers as a fundamental component in a precision medicine framework in the treatment of the most aggressive cancers.

Metabolic rewiring of cancer cells occurs through diverse pathways in a highly context-dependent manner (8–14). However, pan-cancer analyses have identified that some metabolic changes are common among different tumor types with distinct etiologies, despite being mediated by myriad transcriptional changes (15, 16). Understanding the etiology of cancer biomarkers and the implications of this metabolic rewiring in tumor progression may further reveal therapeutic opportunities for exploiting these metabolic dependencies to more effectively target aggressive disease (4, 17). For example, argininosuccinate synthase 1 (ASS1), and ornithine transcarbamylase (OTC) expression status have been used to identify patients who are likely to benefit from arginine-targeted therapies, including arginine deiminase and PEG-arginase therapies, which are showing great promise in improving the sensitivity of a subset of lung and liver tumors to standard-of-care therapies (18–20). Specific companion biomarkers for arginine auxotrophic tumors will be important in identifying patients who will most benefit from this therapy (20).

**Figure 1:**
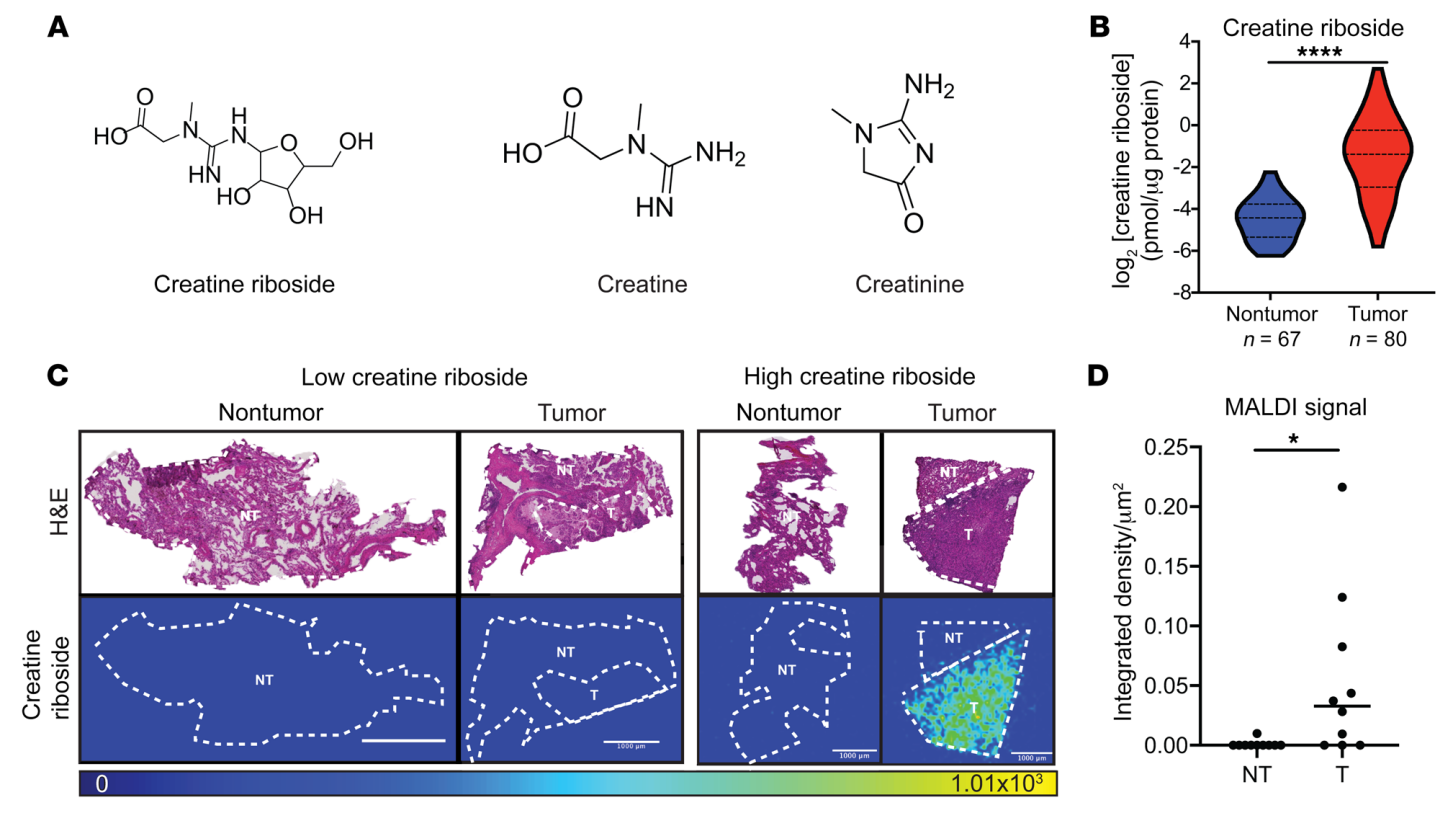
CR is enriched in tumors. (**A**) The chemical structure of CR, creatine, and creatinine. (**B**) The CR concentration was significantly elevated in tumor (*n =* 80) compared with nontumor (*n =* 67) lung tissue. ****P < 0.0001, by Mann-Whitney *U* test. (**C**) Representative images of CR distribution in human NSCLC tumor (T) and matched adjacent nontumor (NT) tissue measured by MALDI imaging MS. CR^lo^ and a CR^hi^ tumors are shown with H&E staining after imaging in the top panel, and MALDI imaging MS signal of CR distribution within the tissue sections is shown in the lower panel. The MALDI imaging MS signal is pseudocolored to indicate CR abundance (range, 0 to 1.01 × 10^3^). Scale bars: 1000 μm. (**D**) CR enrichment in tumoral compared with nontumoral regions of the lung tissue as measured by the integrated CR signal intensity with MALDI imaging MS. *n =* 10 matched tumor and nontumor samples. *P < 0.05., by Mann-Whitney *U* test.

The metabolite creatine riboside (CR) (Figure 1A) was first identified in an untargeted metabolomics analysis of urine samples from patients with non–small cell lung cancer (NSCLC) and population controls, in whom elevated levels of this metabolite were associated with poor patient survival, particularly in patients with early-stage disease (21). This recently identified metabolite is also associated with an increased risk of developing lung cancer upon, and prior to, a lung cancer diagnosis (22), suggesting that it may result from early metabolic changes.

Recently, urinary CR has also been identified as a biomarker of risk and prognosis in adrenocortical (23) and liver (24) cancer. The metabolic reprogramming of these different tumor types remains diverse (4, 17), yet current data suggest that CR may act as a surrogate marker for a common metabolic program associated with tumorigenesis and tumor progression. However, because the etiology of CR was hitherto unknown, it was unclear whether the metabolic reprogramming associated with CR may be exploited for therapeutic benefit. By combining a multiomics analysis with functional studies in lung and liver cancer, this study reveals CR as a tumor-derived biomarker of arginine dependence and poor prognosis in patients with these diverse cancer types. CR-associated metabolism supports cancer cell proliferation and defines tumors with reduced macrophage and CD8^+^ cell infiltration. The metabolic vulnerabilities of CR^hi^ tumors may be targeted by existing and developing therapies in patients with a poor prognosis across multiple cancer types.

## Results

### CR is a tumor-derived biomarker.

Previously, we had shown that CR levels (Figure 1A) were increased in the urine of patients with NSCLC (21) and increased with tumor size (22). However, CR concentrations could not be precisely quantified in different biospecimens because of the lack of a synthetic standard for this metabolite. We therefore synthesized an analytical standard and developed a precise liquid chromatography–tandem mass spectrometry (LC-MS/MS) assay (25) to quantify the level of CR in NSCLC tumor tissue. Using this assay, we confirmed that the CR concentration was significantly higher in tumor tissue compared with that in adjacent nontumor tissue (*P <* 0.001, Figure 1B). This more precise LC-MS/MS assay confirmed the significant association of high urinary CR levels with poor prognosis in patients with lung cancer (log-rank *P =* 0.017, Supplemental Figure 1A; supplemental material available online with this article; https://doi.org/10.1172/JCI157410DS1) and showed a significant positive correlation of tumoral CR with matched urinary concentrations from the same patient (Spearman’s correlation *r* = 0.6, *P =* 0.006, Supplemental Figure 1B), indicating that urinary CR concentrations reflect intratumoral CR concentrations. In contrast, there was no association between tumoral CR levels and the clinicodemographic factors associated with lung cancer outcomes (26) (Supplemental Table 1).

To confirm that CR was enriched in tumor tissue, we spatially mapped CR levels in NSCLC tumor and matched nontumor tissue sections using matrix-assisted laser desorption/ionization (MALDI) imaging MS (Figure 1, C and D, and Supplemental Figure 1C). This confirmed that CR was significantly enriched within tumor tissue compared with levels in immediately adjacent (Supplemental Figure 1G) as well as noninvolved, nontumor (Figure 1, C and D) tissue, thus significantly correlating with our quantitative LC-MS/MS assay (Supplemental Figure 1, D and E) and further supporting the assertion that urinary CR is a surrogate measure of the intratumoral CR concentration. Importantly, CR was not differentially abundant in inflamed, necrotic, or mucinous regions of lung tissue (Supplemental Figure 1F).

**Figure 2:**
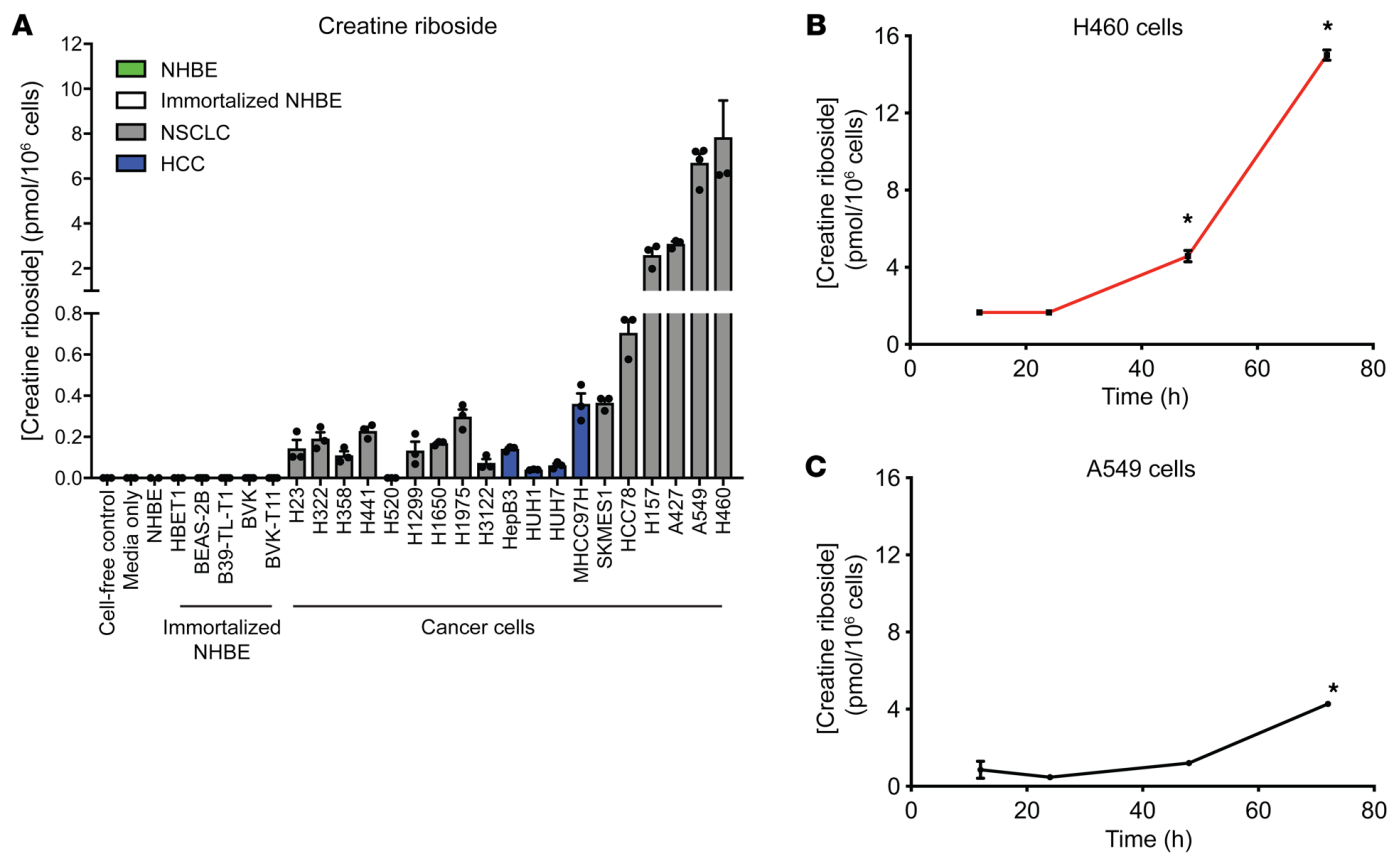
CR is enriched in cancer cells. (**A**) CR was detectable by LC-MS/MS at higher concentrations in cancer cells compared with primary normal and immortalized cells. NHBE, normal human bronchial epithelial cells (green); immortalized NHBE, immortalized normal human bronchial epithelial cells (white); NSCLC cells (gray); HCC cells (blue). Data indicate the mean ± SD of 3–4 independent experiments. (**B** and **C**) Intracellular CR concentrations in H460 (**B**) and A549 (**C**) cell lines grown over time from the time of plating (*t* = 0 h), as measured by LC-MS/MS. Data indicate the mean ± SD of 3 independent experiments. *P < 0.05 compared with the 12-hour time point for that cell line, by Kruskal-Wallis test.

The observation of tumor-enriched CR was further validated with the analysis of human cancer cell lines. We detected higher levels of CR in human NSCLC and hepatocellular carcinoma (HCC) cell lines compared with normal and immortalized nontumorigenic primary human bronchial epithelial cells (Figure 2A). Importantly, CR was not detected in conditioned media or cell-free samples (Figure 2A), indicating that CR is likely a product of cellular metabolism. We found no association between CR levels and intracellular concentrations of creatine or creatinine (Supplemental Figure 1, H and I) or with intracellular concentrations of creatinine riboside, the low-abundance cyclized form of CR (Supplemental Figure 1J). The median intracellular CR concentration (0.213 pmol/10^6^ cells) was used to stratify NSCLC and bronchial cell lines into CR^hi^ and CR^lo^ cell lines (Supplemental Figure 1K) for further interrogation of the metabolic characteristics associated with high CR levels.

To understand the intracellular kinetics of CR production, we monitored the levels of the metabolite in CR^hi^ cells over time. We found that the intracellular CR concentration increased over time, with a significant increase in the levels only after at least 1 population-doubling period (at *t* = 48 h) (Figure 2, B and C), suggesting that a complete cell cycle occurred before CR levels began to increase. Supplementation of CR^lo^ and CR^hi^ cell lines with CR itself did not alter their proliferation rate (Supplemental Figure 2, A–D), despite the detection of extracellularly supplied CR within the intracellular metabolite pool (Supplemental Figure 2E), indicating that CR is a cancer cell–derived biomarker that accumulates with cell proliferation but does not itself induce functional effects.

### CR is formed from creatinine.

**Figure 3:**
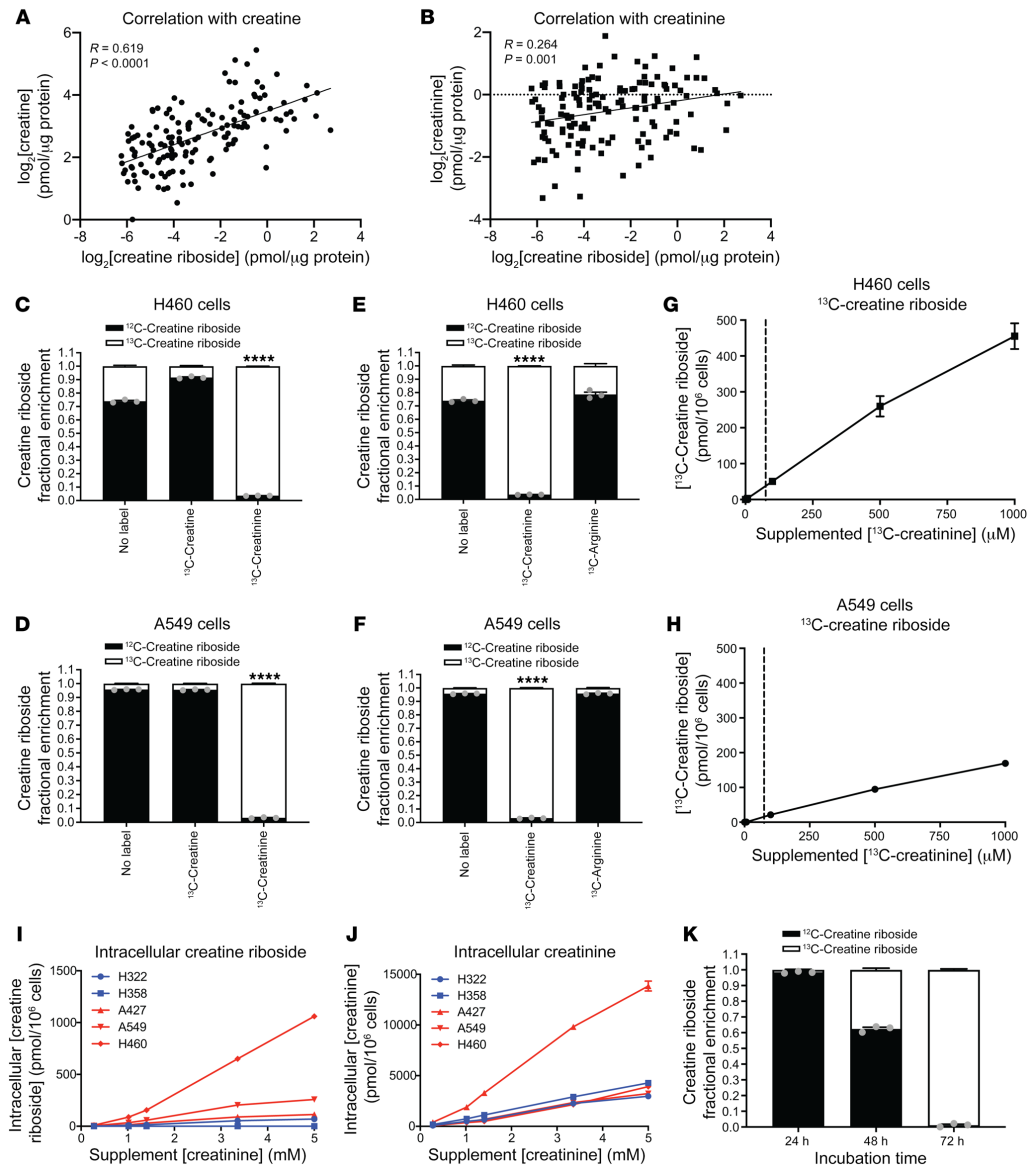
Creatinine is a metabolic precursor of CR. Correlation of CR with creatine (**A**) and creatinine (**B**) within NSCLC tumor tissues (Spearman’s correlation; *n =* 147 tissue samples). (**C** and **D**) Fractional enrichment of CR labeling from ^13^C-creatine or ^13^C-creatinine in H460 (**C**) and A549 (**D**) cells. Data indicate the mean ± SEM of 3 independent experiments. ****P < 0.0001, by 1-way ANOVA with Holm-Šidák correction for multiple comparisons. (**E** and **F**) Fractional enrichment of CR labeling from ^13^C-creatinine or ^13^C-arginine in H460 cells (**E**) and A549 cells (**F**). Data indicate the mean ± SEM of 3 independent experiments. ****P < 0.0001, by 1-way ANOVA with Holm-Šidák correction for multiple comparisons. The ^13^C-creatinine treatment group is replicated from **C** and **D**. (**G** and **H**) CR levels increased with increasing creatinine supplementation as measured by the fractional enrichment of CR labeling from ^13^C-creatinine in H460 (**G**) and A549 (**H**) cells. Dotted line indicates endogenous serum levels of creatinine (75 μM) in humans. Data indicate the mean ± SEM of 3 independent experiments. (**I**) Intracellular CR concentrations in CR^lo^ (blue) and CR^hi^ (red) cell lines with increasing concentrations of exogenously supplied creatinine. Data indicate the mean ± SEM of 3 independent experiments (**J**) Intracellular creatinine concentrations in CR^lo^ (blue) and CR^hi^ (red) cell lines with increasing concentrations of exogenously supplied creatinine. Data indicate the mean ± SEM of 3 independent experiments. (**K**) Time course of the fractional enrichment of CR labeling from ^13^C-creatinine over 72 hours in H460 cells. Data indicate the mean ± SEM of 3 independent experiments.

Structurally, CR is a ribosylated form of creatine, leading us to hypothesize that CR may reflect dysregulated creatine metabolism within lung and other tumors. In healthy tissues, creatine is synthesized from arginine in a 2-step pathway, with the first step being the production of guanidinoacetate in the kidneys through l-arginine:glycine amidinotransferase (AGAT) activity, followed by its conversion to creatine by guanidinoacetate *N*-methyltransferase (GAMT) expressed in hepatocytes (27). Creatine is released systemically to be taken up via the creatine transporter SLC6A8 into other cells, such as lung epithelia, which lack the expression of AGAT and GAMT necessary for creatine synthesis. Intracellular creatine can then be enzymatically phosphorylated to phosphocreatine or spontaneously and passively cyclized to creatinine and excreted (27). Quantitative analysis of tumor and adjacent nontumor lung tissue revealed that tissue CR levels positively correlated with both creatine and creatinine levels (Figure 3, A and B), suggesting that CR levels reflect creatine metabolic dysregulation in tumors. The concentration of creatine and creatinine in tumor tissue exceeded that of CR by approximately 30- and 5-fold, respectively, indicating that CR constituted a relatively small proportion of the total creatine metabolite pool. These data support the notion that CR is a tumor-derived metabolite and potential biomarker of dysregulated creatine metabolism.

Creatine and creatinine emerged as strong candidate metabolic precursors for the creatine moiety of CR because of their structural similarity (Figure 1A) and correlation with CR levels (Figure 3, A and B). To understand the metabolic synthesis of CR, heavy carbons were traced from ^13^C-creatine and ^13^C-creatinine supplied in cell culture media to the intracellular CR pool. Despite the intracellular abundance of exogenously supplied ^13^C-creatine or ^13^C-creatinine, only ^13^C-creatinine, and not ^13^C-creatine, resulted in enrichment of the heavy carbon label within CR (Figure 3, C and D), indicating that creatinine was the precursor for CR.

To determine whether the creatinine that formed CR was synthesized from arginine or imported from the extracellular environment, NSCLC cells were cultured in media containing either ^13^C-creatinine or its biosynthetic precursor ^13^C-arginine, and the incorporation of the heavy carbon label into CR was monitored by LC-MS/MS. CR was labeled when cells were cultured in ^13^C-creatinine but not ^13^C-arginine (Figure 3, E and F), revealing that creatinine biosynthesis was not a major contributor to the CR pool but that instead creatinine imported from the external environment was the CR precursor.

When we extended this analysis to creatinine concentrations below and above (5 μM–1 mM) the physiological plasma concentration of creatinine (75 μM) (28) using ^13^C-creatinine, we found a positive linear relationship between ^13^C-CR and ^13^C-creatinine levels in CR^hi^ cell lines (Figure 3, G and H), confirming the direct conversion of creatinine into CR. We observed similar results in CR^hi^ (Figure 3I) and CR^lo^ (Supplemental Figure 2F) cell lines supplemented with up to 5 mM ^12^C-creatinine, despite comparable concentrations of intracellular creatinine between CR^hi^ and CR^lo^ cells (Figure 3J). These findings thus confirmed that CR was derived from creatinine and that both tumorigenic and nontumorigenic cell lines, regardless of their endogenous CR levels, were capable of producing CR. Importantly, cells with high endogenous CR levels more readily converted creatinine to CR.

The conversion of creatinine to CR was not immediate but occurred gradually over time following 1 complete cell cycle (doubling time was ~24 h) (Figure 3K). This resembled the rate of increase in CR levels with each population doubling (Figure 2, B and C), indicating that creatinine ribosylation, and not creatinine availability, was the rate-limiting step for the conversion of creatinine to CR.

### The ribosylation of creatinine from pentose phosphate pathway products is the rate-limiting step in CR formation.

**Figure 4:**
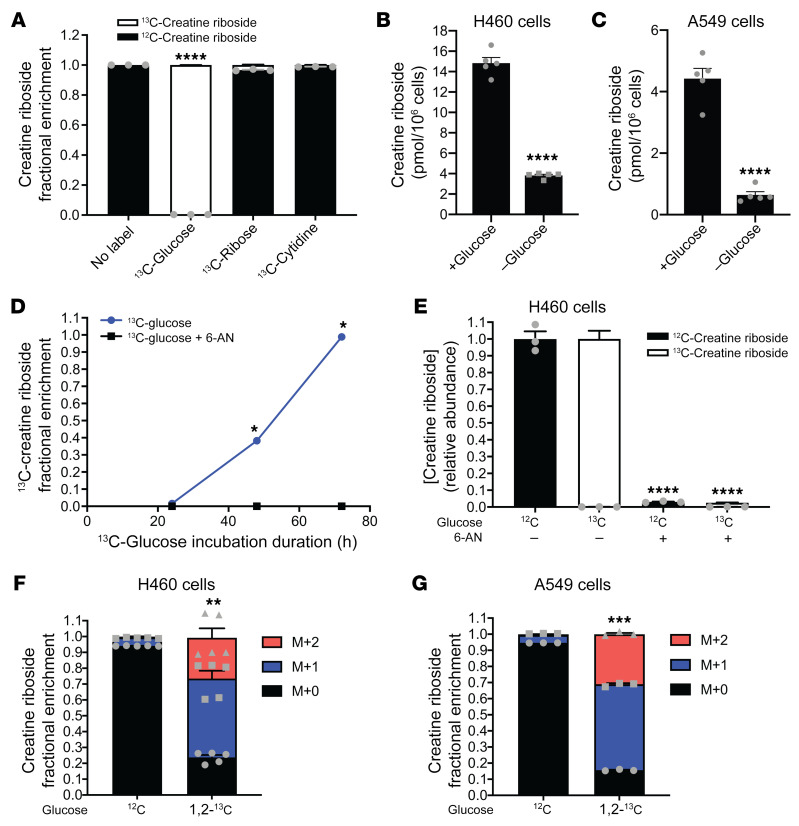
PPP products are the metabolic precursors of CR. (**A**) Fractional enrichment of CR labeling from ^13^C-glucose, ^13^C-ribose, or ^13^C-cytidine in H460 cells. Data indicate the mean ± SEM of 3 independent experiments. ****P < 0.0001, by 1-way ANOVA with Dunnett’s multiple-comparison correction. (**B** and **C**) CR levels in H460 (**B**) and A549 (**C**) cells grown under normal culture (+Glucose) conditions or under glucose starvation (–Glucose). Data indicate the mean ± SEM of 5 independent experiments. ****P < 0.0001, by Mann-Whitney *U* test. (**D**) Fractional enrichment of CR labeling from ^13^C-glucose over time in H460 cells treated with (black line) or without (blue line) the PGDH inhibitor 6-AN. Data indicate the mean ± SEM of 3 independent experiments. *P < 0.05, by 2-way ANOVA with Holm-Šidák multiple-comparison correction. (**E**) Relative abundance of unlabeled and labeled CR in the presence and absence of ^12^C-glucose (^12^C) or ^13^C-glucose (^13^C) and 6-AN in H460 cells. Data indicate the mean ± SEM of 3 independent experiments. ****P < 0.0001, by 1-way ANOVA with Dunnett’s multiple-comparison correction. (**F** and **G**) Fractional enrichment of CR labeling from unlabeled glucose (^12^C) or 1,2 -^13^C_2_-glucose in H460 (**F**) and A549 (**G**) cells. Data indicate the mean ± SEM of 3 independent experiments. ***P < 0.001, by Mann-Whitney *U* test comparison of M + 0 levels.

Ribose itself or ribose intermediates derived through the pentose phosphate or nucleotide metabolism pathways are major sources of ribosylation substrates in cells. Culturing cell lines in ^13^C-glucose but not ^13^C-ribose or ^13^C-cytidine resulted in enrichment of the ^13^C label in CR (Figure 4A), indicating that ribose products derived from glucose metabolism through the pentose phosphate pathway (PPP) were the likely precursors of CR. Consistently, we found that glucose starvation abrogated CR production (Figure 4, B and C). As expected, the rate of ^13^C-CR formation from ^13^C-glucose increased progressively after 1 complete cell cycle (Figure 4D), and resembled the time course of CR formation from creatinine (Figure 2, B and C, and Figure 3K), confirming that the ribosylation of creatinine was the rate-limiting step in CR formation.

To confirm that PPP activity is required for CR formation, cells were treated with 6-aminonicotinamide (6-AN), a competitive inhibitor of 3-phosphoglycerate dehydrogenase (PHGDH), which catalyzes the rate-limiting step of the PPP, and the levels of CR were monitored by LC-MS/MS. Inhibition of PHGDH with 6-AN markedly reduced the enrichment of ^13^C within CR (Figure 4E and Supplemental Figure 3A), as well as the production of endogenous ^12^C-CR (Figure 4E and Supplemental Figure 3A), validating the source of the ribose moiety as a product of the PPP.

Both the oxidative and nonoxidative arms of the PPP can contribute to the formation of ribose intermediates. Culturing cells in 1,2-^13^C_2_-glucose resulted in (M+1) and (M+2) labeling of the intracellular ribose pool and formed both (M+1) CR and (M+2) CR, with (M+1) CR predominating (Figure 4, F and G). This indicates that, while both the oxidative and nonoxidative PPPs produced the precursor to the ribose moiety of CR, the oxidative PPP was the major producer of the ribose moiety precursor. The oxidative PPP culminates in the production of ribose-5 phosphate, which is activated for conjugation with other metabolites or is converted to phosphoribosyl pyrophosphate (PRPP) for ribonucleotide production and is the likely ribosylation substrate in the formation of CR.

Incubation of creatinine and PPP products in human urine samples under various conditions did not produce CR (Supplemental Figure 3B), confirming that CR was produced through an intracellular catalyzed process and was not an extraction artifact.

Together, these data indicate that creatinine and PPP products are the precursors for the metabolite CR and that the conjugation of PPP metabolites with creatinine is the rate-limiting step in the production of CR.

### CR is associated with altered nitrogen metabolism.

**Figure 5:**
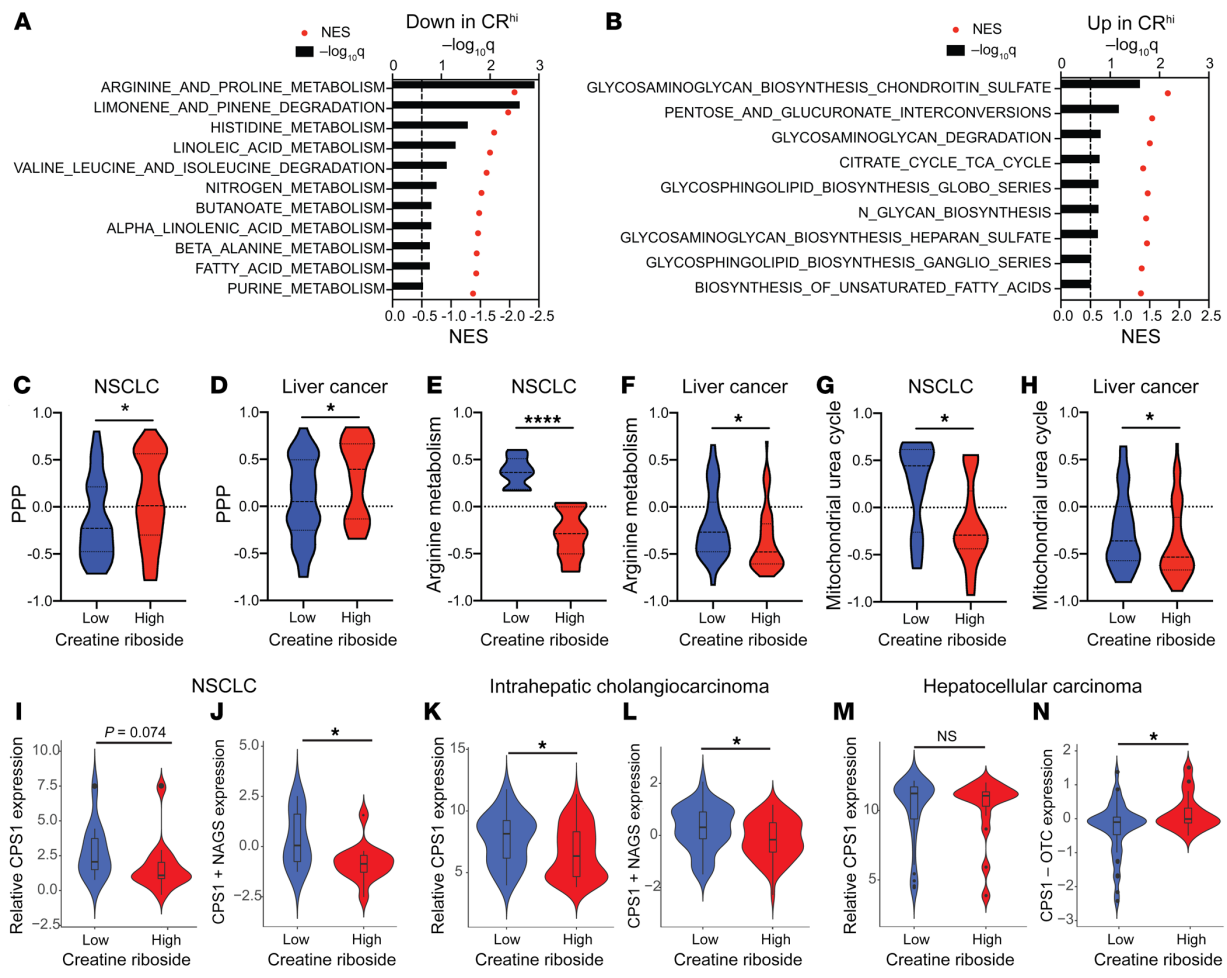
CR is associated with activation of the PPP and urea cycle dysfunction. (**A** and **B**) GSEA of non–small cell lung tumor transcriptional data identified metabolic pathways that were downregulated in CR^hi^ tumors (**A**) and enriched in CR^hi^ tumors (**B**) compared with CR^lo^ tumors. Black bars: –log_10_(*P* values were adjusted for multiple comparisons); red dots: normalized enrichment score (NES). (**C**–**H**) Pathway GSVA of pentose phosphate (**C** and **D**), arginine (**E** and **F**), and mitochondrial urea cycle (**G** and **H**) metabolic pathways in CR^hi^ tumors (lung, *n =* 44; liver, *n =* 58) compared with CR^lo^ tumors (lung, *n =* 43; liver, *n =* 33) from lung (**C**, **E**, and **G**) and liver (**D**, **F**, and **H**) cancer. *P < 0.05 and ****P < 0.0001, by Mann-Whitney *U* test. (**I**–**N**) CR^hi^ tumors had dysregulated expression of mitochondrial urea cycle enzymes. Downregulation of CPS1 expression as well as that of its cofactor NAGS was seen in NSCLC (**I** and **J**) and intrahepatic cholangiocarcinoma (**K** and **L**), while HCC had significant upregulation of CPS1 relative to OTC (**M** and **N**). *P < 0.05, by Mann-Whitney *U* test.

Having established the metabolite precursors for CR, we sought to determine the metabolic processes in tumor cells that would give rise to high CR levels. Having demonstrated a strong positive correlation of urinary and tumoral CR levels, we focused on the association of tumoral CR with the metabolic features of tumors. Gene set enrichment analysis (GSEA) of global gene expression profiles in CR^lo^ and CR^hi^ NSCLC tumors using the Kyoto Encyclopedia of Genes and Genomes (KEGG) database, which contains a wide range of metabolic pathways, identified several significantly altered pathways (Figure 5, A and B, and Supplemental Table 2). These included nonmetabolic pathways such as those for ECM-receptor interactions, focal adhesions, and NK cell cytotoxicity, which were significantly enriched in CR^hi^ tumors, and for the spliceosome, which was significantly enriched in CR^lo^ tumors (Supplemental Table 2). Of all the metabolic pathways, the pathway for downregulation of arginine and proline metabolism was identified as the most significantly altered metabolic pathway in CR^hi^ tumors (Figure 5A and Supplemental Figure 4A). Urea cycle (*CPS1*, *NAGS*, *ASS1*) and creatine metabolism (*CKM*, *GATM*) enzymes, which are directly interconnected to arginine synthesis and catabolism (5), were identified as the most significantly differentially expressed genes in this pathway, and leading-edge analysis found that these genes overlapped with other significantly downregulated metabolic pathways, namely those for limonene and pinene metabolism and nitrogen metabolism and histidine metabolism (Figure 5A). Together, this suggests that the altered expression of these genes is central to the metabolic features of CR^hi^ tumors.

In accordance with CR being a product of the PPP (Figure 4), we found that CR^hi^ tumors were also enriched for the pentose interconversion pathway, with the core enriched genes including the PPP enzyme genes *RPE* and *XYLB* and the genes in the UGT family of enzymes, including *UGT1A8*, *UGT1A3*, and *UGDH* (Figure 5B and Supplemental Figure 4E). Upregulation of *XYLB* in CR^hi^ tumors also contributed to upregulation of the glucuronate biosynthesis and glycosaminoglycan synthesis pathways in CR^hi^ tumors (Figure 5B).

Using gene set variation analysis (GSVA) of the core enriched genes from the PPP (*XYLB*, *RPE*, and *TALDO1*) and arginine metabolism (*CPS1*, *NAGS*, *ASS1*, *CKM*, *AOC1*, and *GATM*), together with the rate-limiting enzymes of the PPP (*G6PD* and *PRPS1*), we confirmed that the PPP was upregulated (Figure 5, C and D) and that arginine metabolism was downregulated (Figure 5, E and F) in CR^hi^ tumors compared with CR^lo^ tumors in both the lung and liver tumors (Supplemental Tables 3 and 4). Applying the PPP signatures to the lung adenocarcinoma, squamous NSCLC, and HCC The Cancer Genome Atlas (TCGA) cohorts confirmed that the PPP was significantly upregulated in tumor tissue compared with nontumor tissue across all cancer types (Supplemental Figure 4, H–J).

Importantly, in the NCI-MD cohort, genes of the mitochondrial component of the urea cycle, namely *CPS1*, *NAGS*, and *OTC*, were the most significantly differentially expressed genes of the arginine metabolism pathway in CR^hi^ tumors, and the mitochondrial component of the urea cycle pathway was identified as significantly downregulated in CR^hi^ lung and liver tumors (Figure 5, G and H). Metabolic flux through the urea cycle relies on activity of both the mitochondrial and cytosolic components of the cycle. A focused examination of urea cycle enzyme expression across lung and liver cancer subtypes revealed repression of the mitochondrial urea cycle mediated through different gene expression programs that functionally downregulate mitochondrial urea cycle activity. In NSCLC and intrahepatic cholangiocarcinoma of the liver cancer, the mitochondrial urea cycle enzyme *CPS1* was downregulated either directly (Figure 5, I and K) or via reduced expression of *NAGS*, the enzyme that produces its essential cofactor *N*-acetylglutamate, respectively (Figure 5, J and L, and Supplemental Figure 4B). Projection of the CPS1 plus NAGS expression score onto TCGA squamous NSCLC confirmed that CPS1 plus NAGS expression was significantly lower in tumor compared with nontumor tissue, while a substantial proportion of lung adenocarcinoma tumors have low CPS1 plus NAGS expression compared with nontumor tissue (Supplemental Figure 4, K and L). Conversely, in the HCC subtype of liver cancer, CR^hi^ tumors showed high expression of *CPS1* compared with *OTC* (Figure 5, M and N), a gene expression pattern that prevents continued conversion of carbamoyl phosphate into citrulline to proceed through the urea cycle and overall results in downregulated mitochondrial urea cycle activity (29). Both downregulated CPS1 activity (CR^hi^ NSCLC and intrahepatic cholangiocarcinoma tumors) or OTC activity (CR^hi^ HCC tumors) result in severe arginine auxotrophy (29) and therefore reflect a common repression of mitochondrial urea cycle metabolism in these different cancer subtypes. Consistent with this, we observed downregulation of the mitochondrial urea cycle in tumor tissue compared with nontumor tissue in the TCGA HCC cohort (Supplemental Figure 4M).

In order to validate the metabolic pathways and transcriptional features associated with CR production in additional data sets, we applied these transcriptional signatures to RNA-Seq data from TCGA lung adenocarcinoma, squamous NSCLC, and HCC cohorts and defined CR^hi^-like tumors as those belonging to the highest tertiles for the PPP and for mitochondrial urea cycle dysfunction, whereas CR^lo^-like tumors were defined as those belonging to the lowest tertiles for the PPP and mitochondrial urea cycle dysfunction. GSEA comparing CR^hi^-like and CR^lo^-like tumors recapitulated the enrichment of the PPP, glucuronate interconversions, and depletion of arginine and proline metabolic pathways in CR^hi^ tumors from these different tumor types (Supplemental Figure 4, N–S).

**Figure 6:**
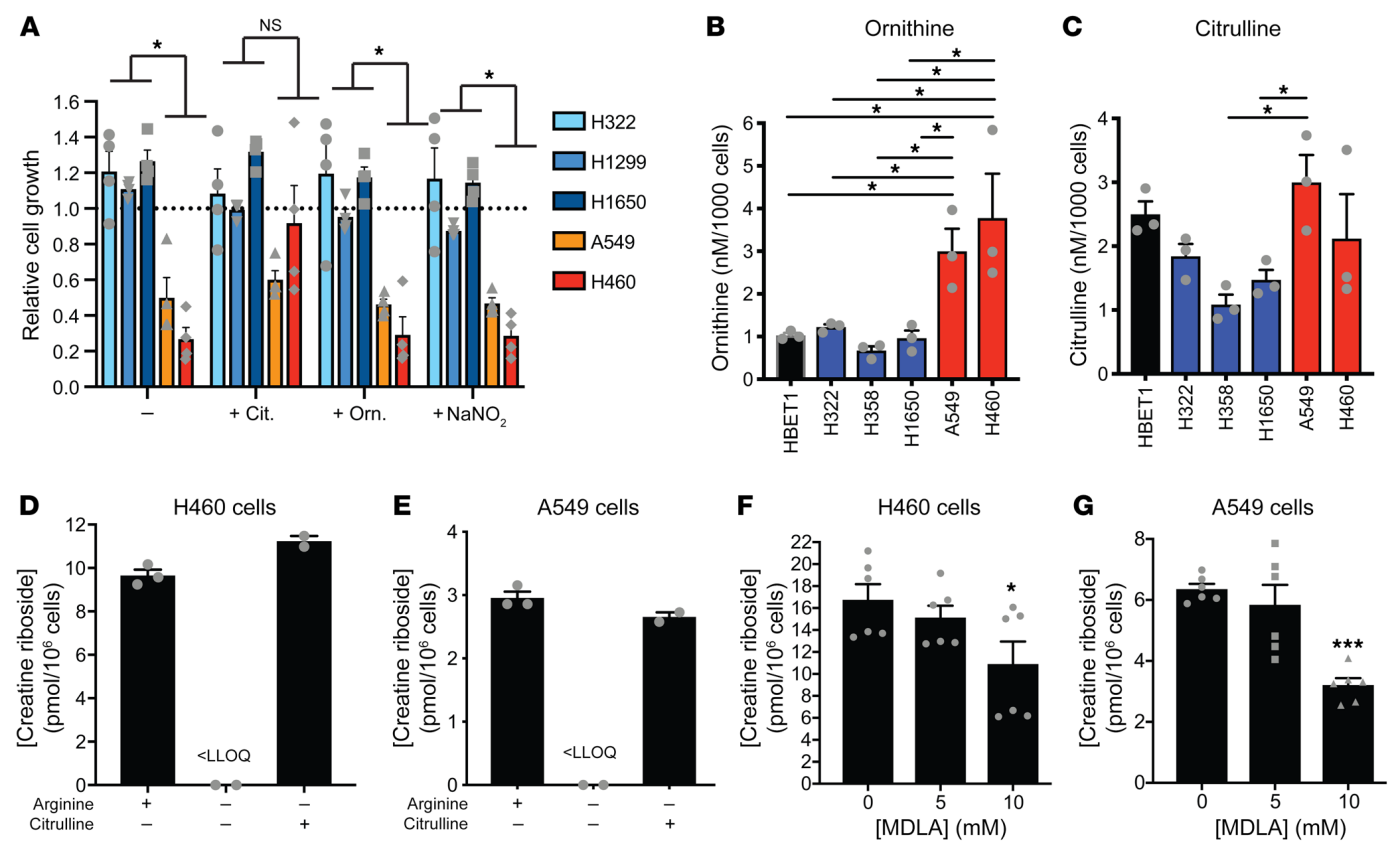
CR is associated with arginine auxotrophy. (**A**) Growth of CR^hi^ (red) and CR^lo^ (blue) NSCLC cell lines in response to arginine deprivation alone (–) or upon supplementation with ornithine (Orn., 1 mM), citrulline (Cit., 1 mM), or sodium nitrite (NaNO_2_). Growth was measured with the MTS assay and is expressed relative to growth in normal culture conditions. Data indicate the mean ± SEM of 4 independent experiments. *P < 0.05, comparing the means of CR^lo^ and CR^hi^, by 2-way ANOVA with Holm-Šidák multiple-comparison correction. (**B** and **C**) Intracellular concentrations of the urea cycle metabolites ornithine (**B**) and citrulline (**C**) in CR^hi^ (red) and CR^lo^ (blue) NSCLC cell lines compared with immortalized bronchial epithelial cells (HBET1, black). Data indicate the mean ± SEM of 3 independent experiments. *P < 0.05, by 1-way ANOVA with Dunnett’s multiple-comparison correction. (**D** and **E**) Arginine deprivation reduced the production of CR in H460 (**D**) and A549 (**E**) NSCLC cell lines. LLOQ, lower limit of quantitation for CR (i.e., lower than the quantifiable limit for the LC-MS/MS assay). Data indicate the mean ± SEM of 2–3 independent experiments. (**F** and **G**) Inhibition of ASS1 with methyl DL-aspartate significantly reduced CR production in H460 (**F**) and A549 (**G**) NSCLC cell lines. Data indicate the mean ± SEM of 6 independent experiments. *P < 0.05 and ***P < 0.001, by 1-way ANOVA with Dunnett’s multiple-comparison correction.

Consistently, CR^hi^ cells (A549, H460), unlike CR^lo^ cells (H322, H1299, H1650), were highly auxotrophic for arginine, as indicated by a significant reduction in cell proliferation when cultured in arginine-free conditions (Figure 6A). Supplementation with citrulline, but not ornithine or the nitric oxide donor sodium nitrite, restored the growth of the CR^hi^ cell lines in these arginine-free conditions (Figure 6A), confirming that the mitochondrial component, rather than the cytosolic component of the urea cycle pathway, was unable to support continued proliferation in the absence of exogenous arginine. Consistent with repression of mitochondrial urea cycle activity, CR^hi^ cell lines had high steady-state levels of ornithine (Figure 6B), whereas citrulline (Figure 6C), arginine (Supplemental Figure 5A), and argininosuccinate (Supplemental Figure 5B) levels were not significantly different compared with C^lo^ cell lines.

In support of the reliance of CR^hi^ cells on arginine metabolism through the cytosolic urea cycle, arginine depletion substantially reduced CR levels in CR^hi^ (Figure 6, D and E) and CR^lo^ cell lines (Supplemental Figure 5C). Under these conditions, citrulline supplementation restored flux through the cytosolic urea cycle, as indicated by elevated levels of argininosuccinate and arginine, and increased CR concentrations to levels in arginine-replete conditions (Figure 5, D and E, and Supplemental Figure 5, C–G). Similarly, inhibition of flux through the cytosolic urea cycle by *N*-methyl-d,l-aspartate (MDLA) (competitive inhibitor of rate-limiting cytosolic urea cycle enzyme ASS1) (5) treatment also significantly reduced CR levels (Figure 6, F and G, and Supplemental Figure 5, H and I), indicating that the ribosylation of creatinine was dependent upon active cytosolic urea cycle metabolism.

Notably, arginine deprivation significantly increased intracellular creatinine concentrations (Supplemental Figure 5, D–G), indicating that arginine starvation reduces CR production by inhibiting creatinine ribosylation, rather than by limiting creatinine availability. Together, this confirms that CR is associated with loss of flux through the mitochondrial component of the urea cycle and renders these cells dependent on exogenous arginine to maintain proliferation.

### The CR metabolic phenotype is characterized by biased nucleotide metabolism.

Recent studies have highlighted that urea cycle metabolism is dysregulated in subsets of multiple tumor types, including NSCLC and liver cancer, for which CR has prognostic importance and has the potential to create multiple metabolic vulnerabilities by inducing changes in amino acid and nitrogen metabolism (5, 30). To further characterize the global metabolic vulnerabilities associated with dysregulated urea cycle metabolism in CR^hi^ NSCLC tumors, we performed global metabolic profiling of the tumor tissues for which intratumoral CR levels had been quantified. Ingenuity Pathway Analysis identified that CR levels were significantly correlated with altered nitrogen metabolism and, in particular, the purine metabolism, amino acid biosynthesis, urea cycle, and oxidative phosphorylation pathways (Figure 6A), as predicted by the transcriptional profiles of these tumors (Figure 4).

**Figure 7:**
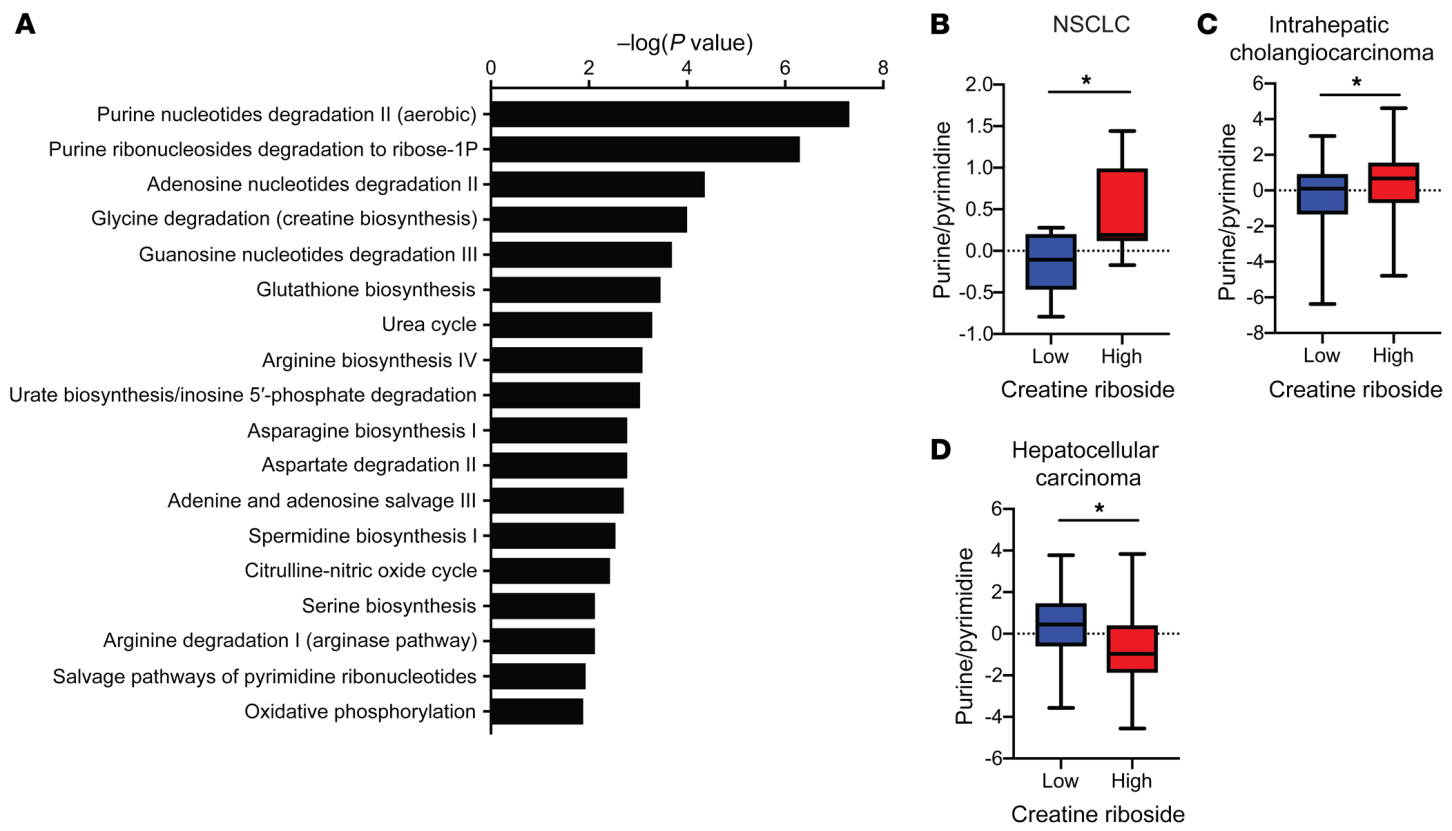
Urea cycle dysregulation in CR^hi^ tumors is associated with a nucleotide pool imbalance. (**A**) Ingenuity Pathway Analysis of metabolic pathways that correlate with metabolite levels in CR^hi^ NSCLC tumors compared with CR^lo^ NSCLC tumors. (**B**–**D**) CR^hi^ tumors had a purine/pyrimidine nucleotide balance that was biased toward purines in NSCLC (**B**, low, *n =* 9; high, *n =* 8) and intrahepatic cholangiocarcinoma (**C**, low, *n =* 49; high, *n =* 75) and toward pyrimidines in HCC (**D**, low, *n =* 42; high, *n* = 17). *P < 0.05, by Mann-Whitney *U* test.

Purine metabolism was the most significantly correlated metabolic change observed in CR^hi^ NSCLC tumors (Figure 7A), supported by significant enrichment of purine compared with pyrimidine metabolites in CR^hi^ NSCLC tumors (Figure 7B). This is consistent with recent studies identifying altered CPS1 expression in driving a purine/pyrimidine bias (4, 29). CR^hi^ intrahepatic cholangiocarcinoma tumors were also enriched for purines compared with pyrimidines (Figure 7C), consistent with their downregulated CPS1 activity that reduced the supply of carbamoyl phosphate to de novo pyrimidine synthesis and enhanced aspartate availability for de novo purine synthesis. Conversely, the low expression levels of *OTC* relative to *CPS1* in CR^hi^ HCCs (Figure 5N) manifested as increased pyrimidine pool sizes compared with purine nucleotide pool sizes, as expected (Figure 7D), reflecting the increased supply of carbamoyl phosphate to carbamoyl-phosphate synthetase 2 (CAD) and activation of de novo pyrimidine synthesis, as predicted from our transcriptional analysis (Figure 5). This observation indicates that the urea cycle dysregulation associated with high CR levels produces a purine/pyrimidine bias and nucleotide imbalance in lung and liver tumors.

**Figure 8:**
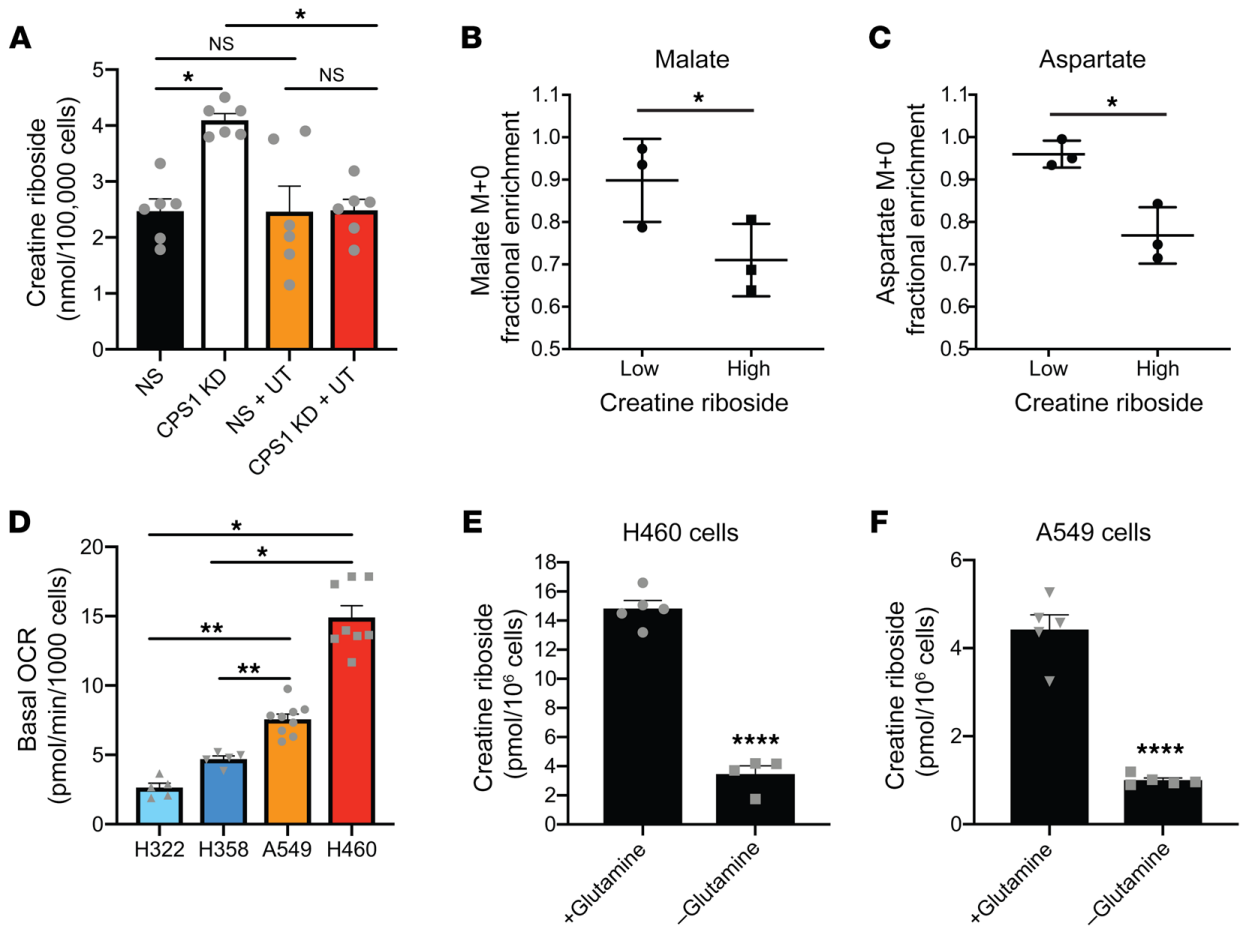
Urea cycle dysregulation drives a nucleotide pool imbalance and high rates of oxidative phosphorylation that promote CR production. (**A**) Suppression of CPS1 expression increased CR levels in normal growth conditions but not when the pyrimidine pools were supplemented. NS, nonsilencing; CPS1 KD, CPS1 knockdown; U, uridine supplementation; T, thymidine supplementation. Data indicate the mean ± SEM of 6 independent experiments. *P < 0.05, by 1-way ANOVA with Dunnett’s multiple-comparison correction. (**B** and **C**) Fractional enrichment of unlabeled malate (**B**) and aspartate (**C**) from U-^13^C-glucose in CR^hi^ and CR^lo^ NSCLC cell lines. Data indicate the mean ± SEM of 3 independent experiments. *P < 0.05, by Mann-Whitney *U* test. (**D**) Oxygen consumption rate in CR^hi^ (red) and CR^lo^ (blue) NSCLC cell lines. Data indicate the mean ± SEM of 5–8 independent experiments. *P < 0.05 and **P < 0.01, by Mann-Whitney *U* test. (**E** and **F**) CR levels in normal growth conditions (+Glutamine) and in glutamine deprived culture conditions (–Glutamine) in H460 (**E**) and A549 (**F**) cells. Values from normal growth conditions are the same as those presented in Figure 4, B and C. Data indicate the mean ± SEM of 4–5 independent experiments. ****P < 0.0001, by Mann-Whitney *U* test.

Given the central role for CPS1 in regulating carbamoyl phosphate and aspartate availability to balance purine and pyrimidine biosynthesis (29, 30), we hypothesized that the purine-to-pyrimidine nucleotide imbalance induced by CPS1 downregulation may drive CR production in NSCLC tumors. Indeed, suppression of CPS1 expression by RNA interference significantly increased CR levels under normal growth conditions (Figure 8A). Maintenance of pyrimidine pools by uridine and thymidine supplementation abrogated this effect, indicating that downregulation of CPS1 increased CR production through depletion of pyrimidine pools, resulting in a purine/pyrimidine nucleotide imbalance. In this way, CR levels were a surrogate for the activity of the mitochondrial urea cycle in tumors. The precise mechanism by which a CPS1-mediated purine/pyrimidine nucleotide imbalance leads to the ribosylation of creatinine to produce CR remains the subject of ongoing studies.

Metabolomics analysis of CR^hi^ tumors also identified significant dysregulation of oxidative phosphorylation (Figure 7A), which regulates nicotinamide adenine dinucleotide (NAD^+^) levels for nucleotide synthesis (31–33). To understand how central carbon metabolism may be rewired to support purine nucleotide synthesis, we performed ^13^C-glucose tracing experiments, which confirmed that CR^hi^ cell lines had significant enrichment of ^13^C into tricarboxylic acid intermediates (Figure 8B and Supplemental Figure 6, A–D). Importantly, CR^hi^ cell lines had increased aspartate labeling from glucose compared with CR^lo^ cell lines (Figure 8C). Aspartate is a limiting metabolite in nucleotide synthesis (32, 33), such that increased aspartate synthesis from elevated tricarboxylic acid and oxidative phosphorylation activity in CR^hi^ NSCLC tumors would support a purine synthesis bias. This is further supported by increased oxidative phosphorylation in CR^hi^ cell lines compared with CR^lo^ cell lines (Figure 8D). Similarly, depletion of glutamine, a metabolite that enables high tricarboxylic activity through anaplerotic reactions, significantly reduced CR levels (Figure 8, E and F), further indicating a role for high tricarboxylic acid activity in supporting biased nucleotide synthesis in CR^hi^ tumors.

Together, these findings indicate that CR^hi^ tumors have a distinct metabolic phenotype compared with that of CR^lo^ tumors. In CR^hi^ tumors, dysfunction of the mitochondrial urea cycle pathway generated a purine/pyrimidine imbalance that promoted creatinine ribosylation. High rates of tricarboxylic acid cycle and oxidative phosphorylation activity supplied this nucleotide bias, sustaining this metabolic phenotype in CR^hi^ tumors (Supplemental Figure 7).

### The CR metabolic phenotype supports rapid proliferation.

Having established the metabolic remodeling in CR^hi^ tumors, we sought to gain further insight into the potential oncogenic reprogramming underlying this remodeling. Whole-exome sequencing of NSCLC tumors identified driver mutations at a frequency similar to that previously reported (Supplemental Figure 8A) (34), however, there were no driver mutations uniquely associated with CR^hi^ tumors (Supplemental Figure 8B). This is consistent with findings in cell lines, in which CR levels were also not significantly associated with known driver mutations (Supplemental Table 5). Diverse mutational events probably contribute to the high CR phenotype, analogous to the myriad transcriptional changes that converge on individual dysregulated pathways across multiple cancer types (15).

The purine/pyrimidine nucleotide bias we identified in CR^hi^ tumors has the potential to alter antigen presentation in the tumor microenvironment to influence the immunological landscape and response to immune checkpoint therapy (30). Cell-type deconvolution of the bulk RNA-Seq expression data from NSCLC tumors identified a significant reduction in monocytes and CD4^+^ memory resting T cells in CR^hi^ lung tumors (Supplemental Figure 8C). Multiplexed immunofluorescence staining supported these findings, identifying a strong negative correlation between CR levels measured by MALDI imaging MS and the number of CD68^+^ (Spearman’s *r* = –0.72, *P =* 0.023) and CD8^+^ T cells (Spearman’s *r* = –0.65, *P =* 0.049) (Supplemental Figure 8D). Comparatively, there was no significant association of CR levels with PD-1^+^ cells in this analysis (Supplemental Figure 8D). Together, this analysis indicates intrinsic reduction in the level of CD68^+^ macrophages and CD8^+^ T cells in CR^hi^ lung tumors. The reduced levels of tumor-infiltrating macrophages and CD8^+^ T cells may contribute to a worse prognosis in CR^hi^ tumors (35, 36), warranting further interrogation of these relationships in larger cohorts.

**Figure 9:**
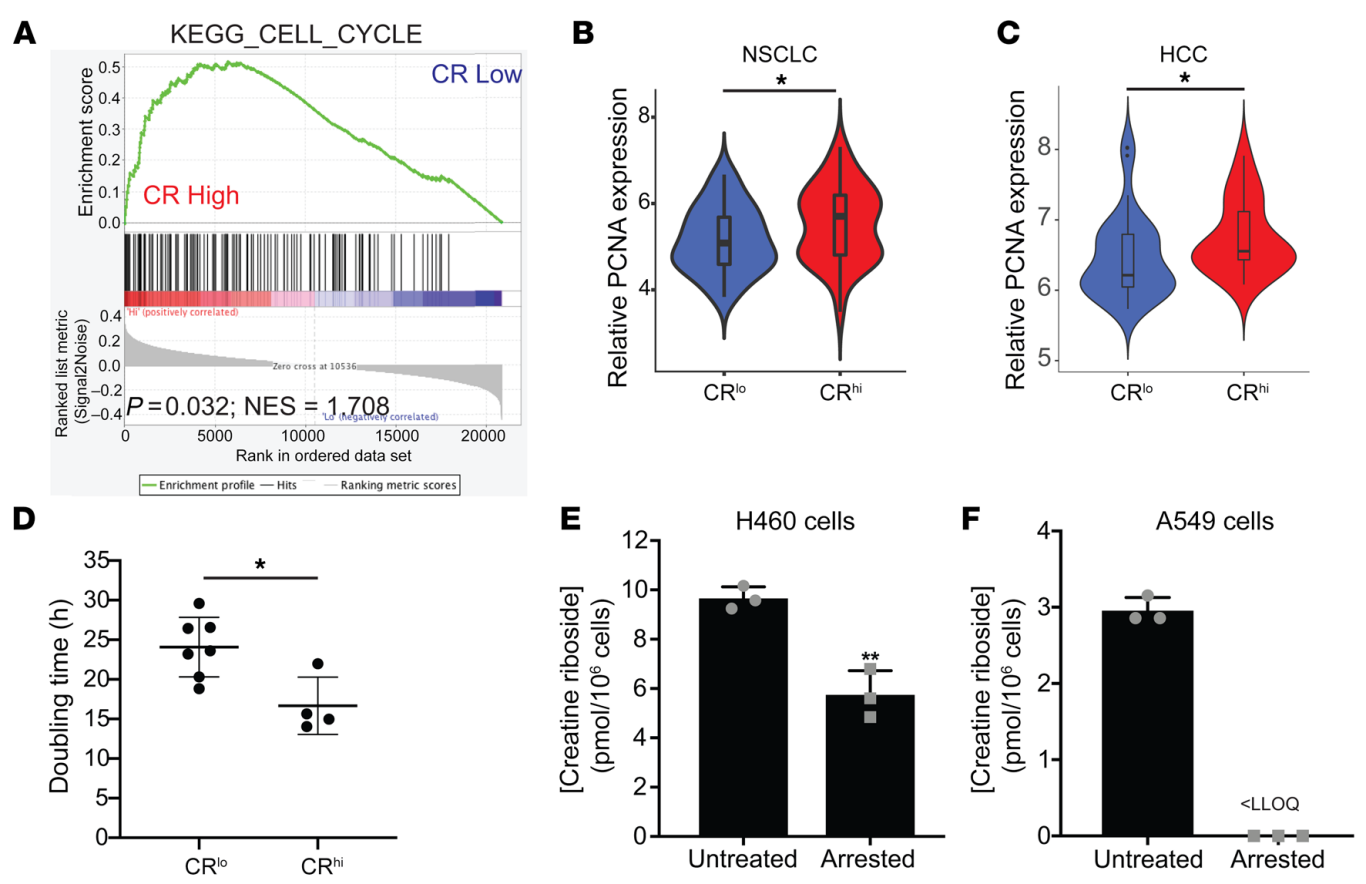
CR is associated with cell proliferation. (**A**) GSEA of NSCLC RNA-Seq data identified that CR^hi^ tumors were significantly enriched in the expression of cell-cycle genes compared with CR^lo^ tumors. *P* values were determined by Kolmogorov-Smirnov statistic with sample randomization. (**B** and **C**) CR^hi^ NSCLC (**B**) and HCC (**C**) tumors had high PCNA expression. *P < 0.05, by Mann-Whitney *U* test. (**D**) Doubling time of NSCLC cell lines as measured by trypan blue dye exclusion and cell counts. Data indicate the mean ± SEM of 2 independent experiments. *P < 0.05, by Mann-Whitney *U* test. (**E** and **F**) Intracellular CR levels in H460 (**E**) and A549 (**F**) cell lines following cell-cycle arrest induced by 2 mM thymidine. Data indicate the mean ± SEM of 3 independent experiments. **P < 0.01, by Mann-Whitney *U* test. LLOQ, lower limit of quantitation, indicating that the CR concentration was lower than the quantifiable limit for the LC-MS/MS assay.

**Figure 10:**
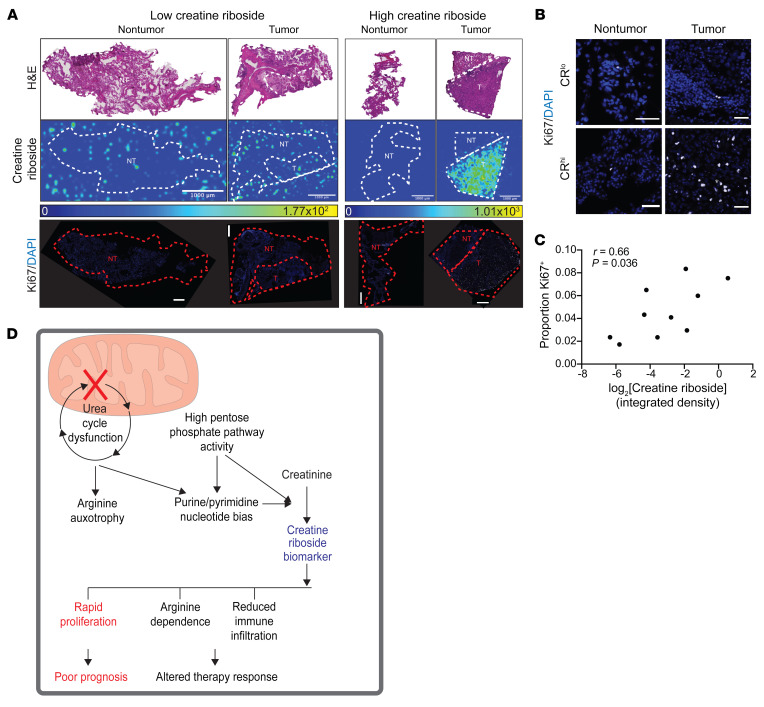
CR^hi^ tumors are highly proliferative. (**A**) Ki67 immunostaining of tumors (DAPI is shown in blue and Ki67 in gray) indicates that CR^hi^ tumors were enriched in Ki67^+^ cells. Scale bars: 1000 μm (MALDI mass spectrometry imaging of creatine riboside) and 500 μm (Ki67 staining). Images are representative of 10 matched tumor and nontumor tissues. (**B**) High-magnification images from **A**. Scale bar: 100 μm. (**C**) Scatter plot shows a correlation (Spearman’s) of the proportion of Ki67^+^ cells relative to the CR signal determined by MALDI imaging MS. (**D**) Schematic representation of the consequences of metabolic rewiring associated with CR^hi^ tumors.

To gain insight into the contribution of the CR-associated metabolic phenotype to tumor progression, we examined nonmetabolic pathways identified as significantly up- and downregulated in CR^hi^ compared with CR^lo^ NSCLC tumors by GSEA. This revealed that CR^hi^ tumors were significantly enriched for cell-cycle–regulating genes (Figure 9A), with core enrichment of regulators of G_1_/S transition (e.g., *CDK6*, *CDC6*, *CCNE1*) and early S-phase progression (e.g., *DBF4*), when nucleotide pool imbalances can induce cell-cycle arrest. This is consistent with a purine/pyrimidine bias supporting the metabolic demands of rapidly proliferating cells (37). The higher proliferation of CR^hi^ tumors was reflected in significantly elevated expression of PCNA (Figure 9, B and C), a marker of cell proliferation that regulates DNA synthesis (38). We found similar results in TCGA adenocarcinoma, squamous NSCLC, and HCC cohorts, with CR^hi^-like tumors enriched for the cell-cycle pathway and significantly higher PCNA expression compared with CR^lo^-like tumors (Supplemental Figure 8, F–K). Similarly, CR^hi^ cell lines had significantly reduced doubling times compared with CR^lo^ cell lines (Figure 9D and Supplemental Figure 6G). Reduced CR levels in cells induced into a state of cell-cycle arrest (Figure 9, E and F) confirmed that CR levels reflect high rates of cell proliferation. To validate these findings in human lung tumors, we performed immunofluorescence staining for Ki67 in the same tumors for which CR levels had been spatially analyzed by MALDI imaging MS (Figure 1C). The number of Ki67^+^ cells was significantly and positively correlated with the spatial CR signal in these lung tumors (Spearman’s *r* = 0.66, *P =* 0.036) (Figure 10, A–C). This confirms that CR was produced at elevated levels in highly proliferative tumors.

Together, these results identify CR as a biomarker of urea cycle dysfunction and nucleotide imbalance in multiple cancer types. This metabolic rewiring drives highly proliferative primary tumors to promote tumor progression, resulting in poor patient survival (Figure 10D).

## Discussion

Metabolic rewiring of cancer cells supports tumor progression and represents a potential source of biomarkers for cancer detection and treatment response. Understanding the origin of metabolic biomarkers enables the personalized and rational design of precision medicine treatments that exploit metabolic vulnerabilities in tumors. This study has revealed that CR, a recently identified prognostic metabolite detected in urine, serum, and tissue biospecimens, is a tumor-derived biomarker of altered urea cycle and PPP activity that promotes tumor growth across multiple tumor types.

Our analysis identifies CR as a tumor-derived metabolite detectable in serum and plasma biospecimens, building on previous studies of CR as a urinary biomarker of risk and prognosis in lung, liver, and adrenocortical carcinoma (21–24). The strong positive correlation of urinary CR levels with tumoral CR levels enabled us to dissect the metabolic profile of CR^hi^ tumors directly at the tissue level. Furthermore, as urinary CR levels strongly correlated with tumoral CR levels, the detection of CR as a diagnostic and prognostic biomarker could be conducted in a minimally invasive manner by analyzing liquid biopsies. In this way, CR levels may assist in identifying patients with NSCLC or liver cancer at high risk of relapse.

Our analysis indicates that CR levels are a surrogate for tumor-associated metabolic reprogramming involving hyperactivation of the PPP coupled with dysfunction of the mitochondrial component of the urea cycle. While the ability of cells to produce CR is not confined to cancer cells, the reprogramming of these metabolic pathways in cancer cells enables them to produce higher levels of this metabolite than nontransformed cells. By integrating the pentose phosphate and urea cycle pathways, CR levels amplify the disparity between tumoral and healthy metabolism, thereby underpinning its potential utility as a sensitive diagnostic and prognostic biomarker (21, 22, 24).

This study reveals that high CR levels reflect a diversion of mitochondrial urea cycle metabolites to support nucleotide synthesis, resulting in urea cycle dysfunction and arginine auxotrophy (30). The urea cycle enzyme CPS1 is central to this metabolic diversion in CR^hi^ tumors. Increased CPS1 expression is associated with dysregulated creatine metabolism and PPP activity in NSCLC (29, 39), supporting the importance of this enzyme in CR production. The high expression of CPS1 relative to that of OTC in CR^hi^ HCC observed in our study concurs with global proteomics analysis of tumor and nontumor tissue (40) and has been shown to result in carbamoyl phosphate diversion to the cytosol to enhance CAD activity (5, 29), which is negatively associated with prognosis (41–43). Furthermore, the association of CR with altered urea cycle activity is supported by recent studies showing that creatinine levels and creatine phosphocreatine pathway activity are regulated by urea cycle activity (44). Urea cycle dysfunction supports anabolic metabolism through the diversion of metabolites toward nucleotide synthesis (5, 30, 37, 45), thus fueling the high rates of cell proliferation associated with high CR levels. A recent study of breast cancer revealed that arginine starvation also suppresses oxidative phosphorylation and inhibits cellular proliferation via nucleotide depletion (46), thereby supporting the association of high CR levels with arginine auxotrophy, a purine/pyrimidine bias, and cancer cell proliferation. CR is also a surrogate for hyperactivation of the PPP. The widespread dysregulation of the pentose and glucuronate interconversion pathways in multiple tumor types, albeit by different transcriptional mechanisms (15), suggests that CR may be useful as a predictive and prognostic biomarker in diverse cancer types extending beyond lung and liver cancers.

At the nexus of the urea cycle, pentose phosphate and nucleotide synthesis pathways, CR reflects highly integrated and coordinated regulation of these metabolic pathways. For example, flux through the cytosolic urea cycle pathway, which remains intact in CR^hi^ tumors, activates glycolysis, and supports the production of ribose 5-phosphate by the PPP (47), while intermediates of de novo synthesis of purine nucleotides, such as 5-aminoimidazole-4-carboxamide riboside (AICA riboside), also stimulate catabolism of glucose to pentose products (48). Notably, PRPS1, which is upregulated in CR^hi^ NSCLC tumors, couples the PPP with de novo purine nucleotide synthesis, thereby directly linking the PPP with the purine nucleotide bias seen in these tumors. It is well established that these metabolic changes support hyperproliferative cancer cell phenotypes (5, 30, 37, 45, 49) and thus underlie the association of CR with aggressive, rapidly proliferating tumors. The importance of cell proliferation in CR production may explain why CR levels are elevated many years before a lung cancer diagnosis (22), when hyperplasia is probably already evident (50–52).

Our rigorous analysis of human biospecimens and in vitro studies failed to find evidence that CR could be spontaneously and passively formed in cells, indicating instead that CR synthesis is likely enzymatically catalyzed, or its formation is driven by electrochemical conditions unique to the intracellular environment. CR production requires cell proliferation, and the compartmentalization of metabolic precursors or enzymatic reactions during specific cell-cycle phases in proliferating cells may explain CR kinetics. We propose a model in which a purine/pyrimidine synthesis bias during cell proliferation increases nucleotide metabolism and thereby increases the availability of activated ribose substrates for the ribosylation of creatinine to form CR. This process may involve enzymes involved in nucleotide synthesis that show altered specificity due to a purine/pyrimidine nucleotide bias. The precise mechanism of creatinine ribosylation remains the subject of ongoing studies in the laboratory.

The association of CR with urea cycle dysfunction and altered nucleotide synthesis has substantial therapeutic implications. The arginine auxotrophy induced by urea cycle dysfunction renders CR^hi^ tumors sensitive to arginine-targeted therapies, which are a rapidly emerging and promising approach in the treatment of NSCLC, breast, pancreatic, and liver cancers, among others (5). The majority of trials testing recombinant human arginase or arginine deiminase therapies have used loss of ASS1 expression as an indicator of therapeutic efficacy (5, 19), however, ASS1 expression levels alone are not always indicative of therapeutic activity (53). Our analysis suggests that CR levels within tumors or liquid biopsies may be a companion diagnostic for arginine auxotrophic tumors that express ASS1 but may also benefit from arginase-based therapies. In this way, a CR biomarker identifies a subset of ASS1-competent tumors that are likely to respond to arginine-targeted therapies. Furthermore, since CR levels reflect the contribution of multiple genes to the dysregulation of the urea cycle, CR levels may identify more cancer patients who are likely to respond to arginase-based therapies compared with the current rationale of using ASS1 expression alone, which fails to identify subsets of tumors that will be sensitive to this emerging therapy (54). Similarly, the association of CR levels with a purine/pyrimidine bias suggests that CR^hi^ tumors are also likely to be sensitive to existing nucleotide imbalance–targeting therapeutics such as methotrexate, whereas pyrimidine synthesis–targeting therapeutics such as pemetrexed and 5-fluorouracil (55) are likely to be more effective in CR^hi^ HCC tumors. The association of high CR levels with reduced macrophage and CD8^+^ immune cell infiltrates suggests that CR levels may not only reflect an altered immunological landscape, as seen in tumors with urea cycle dysfunction (30), but the potential of arginine-targeted therapies to modulate immunotherapy efficacy in lung and other cancers. Further investigations of these findings in larger, immunotherapy-focused patient cohorts are warranted.

Our analysis clearly indicates that CR levels were enriched in tumor compared with nontumor tissue. Our quantitative analytical method allows for sensitive detection at very low concentrations of CR (25) and is therefore capable of detecting very low concentrations of this metabolite in urinary samples from noncancer patients. Our data indicate that noncancerous cells, namely immortalized bronchial epithelial cells, are capable of producing CR when substituted with creatinine and therefore raise the possibility that normal cells may contribute to the low CR signal seen in noncancer patients. However, the conditions under which proliferating epithelial cells would be exposed to the supraphysiological creatinine concentrations required to produce low amounts of CR are unclear. Our data indicate that CR is produced by highly proliferative cells with hyperactivation of the PPP and urea cycle dysfunction, which are active metabolic pathways in many normal cell types. Benign lung nodules and premalignant lesions are both associated with hyperplastic changes in lung tissue (56) and represent potential sources of CR signal in noncancer patients. Importantly, benign lung nodules are detected at relatively high frequency in lung cancer screening trials (57, 58). Furthermore, premalignant lung lesions have a high regression rate, with 66% of lesions spontaneously resolving (59), suggesting that a large proportion of patients with premalignant lesions may never be identified in the population, particularly in populations with a high proportion of current and former smokers, despite the theoretical potential for these lesions to produce detectable amounts of CR. Similarly, our previous analysis of urinary CR in population controls as part of a prospective analysis identified an association of higher CR levels in current compared with former smokers in noncancer controls, although there was no association with pack years (22). This suggests that acute smoke exposure in the lung may contribute to CR production. Tobacco smoke exposure has been associated with altered serum concentrations of arginine, ornithine, and glutamate as well as other lipid metabolites, although the mechanisms underlying these observations remain unknown (60). Therefore, smoking-associated alterations in the levels of these metabolites may modulate the low levels of CR detected in population controls. Tobacco smoke exposure also promotes a proinflammatory response in the lung that activates pneumocyte proliferation (61, 62). Similarly, ROS induced in the lung as a result of tobacco smoke exposure also activate the NFE2-like bZIP transcription factor 2 (NRF2) pathway (63), which has been shown to upregulate the PPP (64), potentially linking tobacco smoke exposure to inflammation-associated metabolic reprogramming that may contribute to CR production. Although CR levels were not higher in inflamed intratumoral regions in our analysis, it remains to be determined whether widespread lung inflammation from acute tobacco smoke exposure may contribute to the very low CR levels detected in noncancer patients. The source of the very low levels of CR detected in noncancer patients is an area of ongoing intense research in the laboratory and will underpin the use of CR as a diagnostic biomarker.

Overall, CR is a prognostic tumor-derived metabolite reflecting concomitant urea cycle dysfunction and PPP hyperactivity that support cancer cell proliferation. These metabolic vulnerabilities may be exploited to identify and more effectively target aggressive CR^hi^ tumors that have the worst prognosis in multiple cancer types.

## Methods

### Materials.

Mass spectrometric standards were purchased from MilliporeSigma and Cambridge Isotope Laboratories. CR was custom synthesized (25).

### Cell culture.

All cell lines were obtained internally from the NCI-60 panel (H460, A549, A427, HCC78, SKMES1, HUH1, HUH7, Hep3B, H3122, H1975, H1650, H1299, H441, H358, H322, H23); were purchased from the American Type Culture Collection (ATCC; normal human bronchial epithelial cells); or were developed in-house (HBET1, Beas-2B, and derivatives ; refs. 65, 66). The MHCC97H cell line was a gift from Xin Wei Wang (NCI, NIH, Bethesda, Maryland, USA). Cell lines were cultured in RPMI 1640 or DMEM (Corning) supplemented with 10% FBS (HyClone) and 2 mM l-glutamine (Gibco, Thermo Fisher Scientific). Cell lines obtained from the ATCC or NCI-60 were directly cultured for use or authenticated by short tandem repeat (STR) profiling (ATCC) if they had been obtained more than 6 months prior to use. All cell lines were tested for mycoplasma contamination every 6 months.

### Liquid biospecimen preparation for metabolomics.

Urine samples were prepared for LC-MS/MS analysis as described previously (24).

### Cell line sample preparation for metabolomics.

Culture media and cells were separately harvested in ice-cold extraction buffer (acetonitrile/H_2_O/methanol [65:30:5, v/v/v], 3 μM DL-2-aminopimelic acid). Cell numbers were recorded in parallel. All samples were centrifuged and supernatants were sonicated (2 min, Bioruptor, Cosmo Bio), freeze-thawed in liquid nitrogen, and filtered (Ostro Protein Precipitation Plate, Waters).

### Tissue sample preparation for metabolomics.

Frozen lung tissues (50–100 mg) were cryo-milled and extracted in cold extraction buffer (acetonitrile/H_2_O/methanol [65:30:5, v/v/v], 3 μM DL-2aminopimelic acid). All samples were centrifuged and supernatants were collected for LC-MS/MS analysis.

### LC-MS/MS metabolomics.

To quantitate the metabolite levels in cancer cells, culture media, or urine, extracts prepared as described above were analyzed by ultraperformance liquid chromatography MS/MS (UPLC-MS/MS).

Metabolite quantitation was performed by multiple reaction monitoring with an Acquity UPLC/Xevo TQ-S Micro System (Waters) using a synthetic CR standard (25). For the measurement of urea cycle intermediates, the mobile phase was A:50 mM formic acid in acetonitrile and B:50 mM formic acid in water, pH 3. Separation was achieved on an Acquity UPLC BEH amide column (50 × 2.1 mm, 1.7 μm, Waters). Metabolite concentrations were calculated using calibration curves of analytical standard solutions (Masslynx, Waters) before being normalized to cell numbers.

CR levels were stratified as high or low by the median for lung tissue samples and liver cancer samples, or by the 75th percentile of the population control (i.e., cancer-free individuals) levels for urine samples from patients with lung cancer. Cell lines were classified into CR^hi^ and CR^lo^ groups by the median value.

To test the association of driver mutations with CR levels in cell lines, mutation data were obtained for each cell line from the Cancer Cell Line Encyclopedia, and χ^2^ tests were performed to assess significant associations.

### Gas chromatography–coupled MS detection of ribose.

Quantitative detection of ribose, glucose, and their heavy-labeled forms was performed using gas chromatography–coupled MS (GC-MS). Cell pellets and cell media were harvested in 70% (v/v) acetonitrile and DL-norleucine (10 μM) followed by homogenization (Precellys Homogenizer, Bertin Instruments). Samples were then centrifuged, and the supernatant was dried, *N*,*O*-bis(trimethylsilyl)trifluoroacetamide (BSTFA) derivatized (MilliporeSigma), and resuspended in 50 μL acetonitrile for analysis (1 μL injection).

d-ribose, U-^13^C_5_d-ribose, d-glucose, and U-^13^C_6_d-glucose concentrations were quantified on an Agilent 6890N gas chromatograph coupled to an Agilent 5973 mass-selective detector using standard curves (Agilent MassHunter Workstation Software). Chromatographic conditions and detection details for each ion are provided in the Supplemental Methods.

### RNA-Seq.

Total RNA was extracted from lung tissues as previously described (67), and RNA-Seq processing was performed as outlined in the Supplemental Methods. Batch correction was performed using the Combat algorithm from the SVA package (68).

For analysis of TCGA data, RNA-Seq and clinicodemographic data on lung adenocarcinoma, lung squamous NSCLC, and HCC were downloaded from https://gdc.cancer.gov/about-data/publications/pancanatlas using survival information from (69). Corresponding mutational information was downloaded from GDAC FireHose (43, 70, 71). Projection of the CR status onto TCGA samples was performed using cancer-type–specific approaches revealed by analysis of the NCI-MD cohorts. For projection of the CR status onto TCGA adenocarcinoma and squamous NSCLC samples, (a) samples were tertile stratified according to expression of CPS1 plus NAGS, and then (b) a PPP expression score was calculated using gene set variation analysis (GSVA). NSCLC samples in the lowest tertile for CPS1 plus NAGS expression and the highest tertile for the PPP score were considered CR^hi^-like. NSCLC samples in the highest tertile for CPS1 plus NAGS expression and the lowest tertile for the PPP score were considered CR^lo^-like. For projection of the CR status onto TCGA HCC samples, (a) samples were tertile stratified according to mitochondrial urea cycle activity using GSVA and the mitochondrial urea cycle signature of *CPS1*, *OTC*, and *NAGS*, and then (b) a PPP expression score was calculated using GSVA and the PPP signature *G6PD*, *TALDO1*, *PRPS1*, and *RPE*. HCC samples in the lowest tertile for mitochondrial urea cycle activity (i.e., highest in mitochondrial urea cycle dysfunction) and the highest tertile for the PPP score were considered CR^hi^-like. HCC samples in the highest tertile for mitochondrial urea cycle activity (i.e., lowest in mitochondrial urea cycle dysfunction) and the lowest tertile for the PPP score were considered CR^lo^-like.

For NCI-MD cohort analyses, differential gene expression analysis was performed using EdgeR (72) and Limma (73). Enriched pathways were identified using GSEA against the KEGG subset of the C2 MSigDb collection (74). Cell-type deconvolution was performed using Cibersortx and the LM22 gene signature set of hematopoietic cell subsets in bulk tissues (75, 76).

### Whole-exome sequencing.

Total DNA was extracted from lung tumor tissue and cell lines as described previously (67) (see also Supplemental Methods). Driver mutations and COSMIC signatures were identified using Maftools (77). Transcriptional strand mutations were annotated using MutationalPatterns (78). The transcription-associated purine or pyrimidine transversion mutation bias was calculated by measuring the frequency of purine and pyrimidine transversion mutations on the transcribed strand, as described previously (30).

### Global metabolomics profiling.

Metabolon conducted the global metabolomics analysis of 25 matched lung tumor and adjacent nontumor samples as described for the liver tumor samples (67) (see also Supplemental Methods). Raw metabolite abundances were median scaled for analysis. Pathway analysis was performed using metabolite correlations with CR levels by Ingenuity Pathway Analysis (QIAGEN).

### Stable isotope tracing.

Metabolic pathway activity in CR^lo^ and CR^hi^ cancer cells was examined by tracing stable heavy carbons from glucose through intracellular metabolite pools. Cells were cultured in phenol red–free RPMI media, 10% dialyzed serum, and U-^13^C-glucose (28). Cells were harvested in 80% aqueous methanol containing 500 nM ^15^N–,^13^C–amino acid mix (Cambridge Isotopes Laboratories; MSK-A2-1.2). Samples were dried with a SpeedVac (Thermo Fisher Scientific), resuspended in water, and analyzed by LC/MS as described previously (32) (see also Supplemental Methods). Relative quantitation of polar metabolites was performed with XCalibur QuanBrowser 2.2 (Thermo Fisher Scientific). For stable isotope tracing analyses, data were corrected for natural abundance using an in-house script (79).

### Imaging MS.

Tumor and adjacent nontumor tissue samples were prepared for in situ spatial measurement of CR levels using imaging MS. Fresh-frozen human lung tumor/nontumor tissue paired sections (12 μm, –20°C; CM 1900 Cryostat, Leica Biosystems) were thaw-mounted onto indium tin oxide–coated glass slides and dried, and 2,5-dihydroxybenzoic acid (DHB) (40 mg/mL, 70% methanol with 0.1% trifluoroacetic acid) was applied (TM Sprayer, HTX Technologies). Reconstructed ion images were generated in positive ion mode with a linear ion trap mass spectrometer equipped with a MALDI source and a nitrogen laser (LTQ XL, Thermo Fisher Scientific) using a targeted MS/MS method optimized for CR (diagnostic ion: *m/z* 132) (Supplemental Methods). Images were acquired at 70 μm spatial resolution using a pinwheel filter.

### Cell proliferation.

Cell proliferation (doubling time) and viability were measured by trypan blue dye exclusion and cell counting, as well as with the CellTiter 96 Aqueous One Assay (Promega), according to the manufacturer’s instructions.

### Respirometry.

The rate of oxidative phosphorylation was measured using the Agilent Seahorse XF24 Analyzer as described previously (80) and normalized to cell numbers using cell counts.

### Cell-cycle analysis.

Cell-cycle arrest induced by thymidine blocking was verified using a propidium iodide flow cytometry kit (Abcam) on a FACSCanto II (BD Biosciences) as described previously (81). Cell-cycle distribution was analyzed using FlowJo software (Tree Star).

### OPAL multiplex immunofluorescence staining and imaging.

Frozen serial sections obtained from MALDI imaging MS analysis were fixed in a formaldehyde glutaraldehyde fixative followed by antigen retrieval (Agilent Technologies, catalog S2367). Sections underwent serial staining for Ki67 (primary Ab: Cell Signaling Technology, catalog 9027; secondary Ab: Opal Polaris 480, Akoya Biosciences); CD8 (primary Ab: Abcam, catalog ab101500; secondary Ab: Opal Polaris 780); CD68 (primary Ab: Agilent Technologies, catalog M0876; secondary Ab: Opal Polaris 620); and programmed cell death 1 (PD-1) (primary Ab: Cell Signaling Technology, catalog 43248; secondary Ab: Opal Polaris 570) as outlined in Supplemental Methods. Slides were imaged on a Vectra Polaris (Precision Medicine Group). Multiplexed images were unmixed using InForm software. Quantitative analysis of cell types was performed in QuPath (version 2.3).

### Data and code availability.

RNA-Seq and whole-exome sequencing data for NCI-MD lung tumors are available in the NCBI’s Gene Expression Omnibus (GEO) database (GEO GSE201221). TCGA and NCI-MD liver sample data sets (GEO GSE76297) were accessed as described above. All analyses were performed using publicly available packages as described above and in the Supplemental Methods.

### Statistics.

All statistical analyses were performed in GraphPad Prism 7 (GraphPad Software) or R (version 3.6.3). Differences between groups were assessed with the Mann-Whitney *U* test for comparison of 2 groups or the Kruskal-Wallis test with Dunn’s correction for multiple comparisons. Two-way ANOVA with Dunnett’s correction for multiple comparisons was used for comparison of 2 or more groups across multiple variables. The Benjamini-Hochberg method was used to correct for multiple comparisons for other analyses. Differences in the distribution of mutations among cell lines and their relationship with high and low CR levels were examined using Fisher’s exact test. Spearman’s tests were used to determine correlations. Survival analysis was performed using the R packages Survminer and Survival. Data presented as box plots show the median, box boundaries indicate the 25th and 75th percentiles, and whiskers show the minimum to maximum values. A *P* value of less than 0.05 was considered statistically significant.

### Study approval.

Lung tumor and nontumor biospecimens from the NCI-MD cohort were collected according to procedures approved by the NCI, NIH, as described previously (21) (IRB OH98-C-N027). Liver cancer biospecimens and data were obtained from the TIGER-LC cohort as described previously (24, 67). Written informed consent from patients was received prior to participation in these studies. The clinicodemographic features of these cohorts are outlined in Supplemental Tables 1, 3, and 4.

## Author contributions

ALP and CCH conceptualized the study. ALP, TO, MR, DB, and SK designed the methodology. ALP, LT, TO, DP, and MR conducted the formal analysis. ALP, LT, TO, YK, DP, TT, ST, JAB, MR, DB, and KWK performed experiments. EB, GTP, FJG, SPH, SA, BMR, XWW, and CCH provided resources. EB curated the data. ALP wrote the original draft of the manuscript. Review and Editing ALP, LT, TO, EB, SK, FJG, SPH, SA, BMR, XWW, and CCH reviewed and edited the manuscript. ALP performed visualization. CCH supervised the study, handled project administration, and acquired funding.

## Supplementary Material

Supplemental data

Supplemental table 2

## Version 1 07/15/2022

Electronic publication
