# Supplementary material for: Creatine riboside is a cancer cell–derived metabolite associated with arginine auxotrophy
Source: J Clin Invest. 2022 Jul 15;132(14):e157410. doi: 10.1172/JCI157410 (PMC9282934; doi:10.1172/JCI157410)
Supplement: Supplemental data [file jci-132-157410-s141.pdf]

Supplemental Information

Creatine Riboside is a Cancer Cell-Derived Metabolite Associated with Arginine Auxotrophy

Parker et al.

Supplemental Table 1. ***Clinicodemographic characteristics of the NCI -MD NSCLC cohort.***

		Tissue Biospecimens				Urine Biospecimens		
		**All**	**CRLow**	**CRHigh**	**P-value**	**All**	**CRLow**	**CRHigh**
**Age**	*mean ± sd*	65.9 ± 9.44	67.0±10.4	64.9 ±8.61	0.32*	64.7±9.67	63.0±9.82	64.8±9.68
**Gender**	*Male*	50	25 (59.5%)	25 (59.5%)	>0.99	234	73 (45.1%)	161 (53.3%)
	Female	34	17 (40.5%)	17 (40.8%)		230	89 (54.94%)	141 (46.69%)
**Stage**	*I*	1	0 (0%)	1 (2.38%)	0.74§	29	9 (5.6%)	20 (6.6%)
	IA	20	10 (23.8%)	10 (23.8%)		85	46 (28.4%)	39 (12.9%)
	IB	31	14 (33.3%)	17 (40.5%)		77	31 (19.1%)	46 (15.2%)
	II	0	0 (0%)	0 (0%)		6	0 (0%)	6 (2.0%)
	IIA	12	7 (16.7%)	5 (11.9%)		12	7 (4.32%)	5 (1.66%)
	IIB	10	5 (11.9%)	5 (11.9%)		33	17 (10.5%)	16 (5.30%)
	III	0	0 (0%)	0 (0%)		23	5 (3.1%)	18 (5.96%)
	IIIA	9	5 (11.9%)	4 (9.52%)		47	9 (5.56%)	38 (12.6%)
	IIIB	1	1(2.38%)	0 (0%)		45	14 (8.6%)	31 (10.3%)
	IV	0	0 (0%)	0 (0%)		89	20 (12.4%)	69 (22.9%)
	Unknown	0	0 (0%)	0 (0%)		18	4 (2.5%)	14 (4.64%)
**Histology**	*Adenocarcinoma*	49	25 (59.5%)	24 (57.1%)	0.30^	232	99 (61.11%)	135 (44.7%)
	Adenosquamous	1	1 (2.38%)	0 (0%)		4	2 (1.22%)	2 (0.66%)

	Bronchoalveolar Carcinoma	2	2 (4.76%)	0 (0%)		5	1 (0.62%)	4 (1.32%)
	NSCLC	3	2 (4.76%)	1 (2.38%)		89	20 (12.3%)	69 (22.8%)
	Large cell carcinoma	0	0 (0%)	0 (0%)		2	1 (0.62%)	1 (0.33%)
	Squamous	26	10 (23.81 %)	16 (38.10 %)		127	38 (23.5%)	89 (29.5%)
	Unknown		2 (4.76%)	1 (2.38%)		1	1 (0.62%)	2 (0.66%)
**Smoking Status**	*Never Smoker*	2	1 (2.38%)	1 (2.38%)		33	11 (6.79%)	22 (7.28%)
	Ever Smoker	35	19 (45.2%)	16 (38.1%)		431	151	280 (92.7%)
	Current Smoker	14	8 (19.0%)	6 (14.3%)	0.95$^{\#}$	202	84 (51.85 %)	118 (39.1%)
	Former Smoker	21	11 (26.2%)	10 (23.8%)		229	67 (41.4%)	162 (34.9%)
	Unknown	47	22 (52.4%)	25 (59.52 %)		0	0 (0%)	0 (0%)
**Pack Years**	*mean ±sd*	47.4±28.3	40.5±27.0	43.7±30.2	0.56*	42.8±48.0	45.0±71.7	41.6±28.0

* Statistical significance tested using Mann-Whitney non-parametric test.

§Fisher’s exact test of early (stage I +II) vs late (stage III+IV)

^ Fisher’s exact test of adenocarcinoma vs squamous NSCLC.

Fisher’s exact test of never vs ever smoker

Supplemental Table 2. ***Gene Set Enrichment Analysis of CR^{High} and CR^{Low} lung tumors from the NCI-MD cohort (Attached as excel spreadsheet).***

Supplemental Table 3. ***Clinicodemographic characteristics of the TIGERLC-Intrahepatic Cholangiocarcinoma cohort.***

		**All (n=91)**	**CRLow n=33 (%)**	**CRHigh n=58 (%)**
**Age**	*mean ± sd*	59.7 ± 8.62	58.1 ± 8.81	60.6 ±8.46
**Gender**	*Male*	54	13 (39.4%)	41 (70.6%)
	Female	30	13 (39.4%)	17 (29.3%)
	Unknown	7	7 (21.2%)	0 (0%)

Supplemental Table 4. ***Clinicodemographic characteristics of the TIGERLC-Hepatocellular Carcinoma cohort.***

		**All (n=58)**	**CRLow n=42 (%)**	**CRHigh n=16 (%)**
**Age**	*mean ± sd*	54.9 ± 10.5	54.6 ± 9.96	55.5 ±12.2
**Gender**	*Male*	46	33 (76.74%)	13 (81.2%)
	Female	12	9 (20.93%)	3 (18.7%)

Supplemental Table 5. ***Association of creatine riboside levels with driver mutations in cancer cell lines.***

**Driver Mutations**	**All Cancer Cell Lines p-value**	**NSCLC Cell Lines pvalue**
EGFR-KRAS axis	0.22	1
TP53	0.16	0.2
PI3K-mTOR	0.073	0.28
CDKN2A	0.095	0.076
ALK Fusion	0.48	0.46
CTNNB1	1	1
ROS1 Fusion	1	1
TERT	1	1

Supplemental Methods

Cell Line Sample Preparation for Metabolomics

Culture media was collected and diluted 1:4 with ice-cold extraction buffer (acetonitrile/H_2O/methanol (65:30:5, v/v/v)) containing 3µM DL-2aminopimelic acid (Sigma) as an internal standard. Cells were washed in PBS and scraped in extraction buffer (acetonitrile/H_2O/methanol (65:30:5, v/v/v)). Cell number was recorded in parallel. All samples were centrifuged (21000g, 15min) and supernatants were sonicated (2min, Bioruptor, Cosmo Bio), freeze-thawed in liquid nitrogen and filtered (Ostro Protein Precipitation & Phospholipid Removal Plate, Waters).

Tissue Sample Preparation for Metabolomics

Frozen lung tissues (50-100mg) were weighed then milled (cryomill, -196°C). Cold extraction buffer (acetonitrile/H_2O/methanol (65:30:5, v/v/v) containing 3µM DL-2-aminopimelic acid (Sigma)) was added directly to the milled tissues. All samples were centrifuged (21000g, 15min) and supernatants were collected for LC-MS/MS analysis.

LC-MS/MS Metabolomics

To quantitate the metabolite levels in either cancer cells, culture media or urine, extracts were prepared as described above and analyzed by UPLCMS/MS.

Metabolite quantitation in extracts of cell lines, culture media, human urine and human lung tissue samples was performed by multiple reaction monitoring (MRM) using an Acquity UPLC/Xevo TQ-S micro system (Waters Corp) following an optimized quantitative protocol using a synthetic creatine riboside standard as previously described (1). Briefly, five microliters of each sample was injected to the system and chromatographic separation was achieved on

an Acquity UPLC BEH amide column (50 × 2.1 mm internal diameter, 1.7 μm, Waters Corp). The mobile phase was a mixture of acetonitrile/H_2O (10/90, v/v, buffer A) and acetonitrile/H_2O (90/10, v/v, buffer B); pH was adjusted at 9.0 for both buffer A and B using ammonium acetate and ammonium hydroxide. Gradients were run starting from 99% buffer B to 60% buffer B from 0–6 min; 60% buffer B to 20% buffer B from 6–8 min; 20% buffer B to 99% buffer B from 8–10 min; and 99% buffer B was held for 2 min to re-equilibrate the column. The column temperature was maintained at 40°C and the flow rate was 0.4 ml/min. For measurement of urea cycle intermediates the mobile phase was a mixture of 50mM formic acid in acetonitrile (Buffer A) and 50mM formic acid in water (Buffer B) at pH 3 with separation achieved on an Acquity UPLC BEH amide column (50 × 2.1 mm internal diameter, 1.7 μm, Waters Corp).

Mass spectrometry data were acquired in the positive electrospray ionization mode. The dwell time was approximately 4 ms per MRM transition, and approximately 10–12 data points were acquired for each detected metabolite. Signal intensities were extracted and processed using TargetLynx software (Waters Corp). Areas under the peak for each metabolite MRM transition were extracted and normalized to those for the internal standard. Metabolite concentrations in the samples were calculated using calibration curves generated from analytical standard solutions, and then intracellular metabolite concentrations were normalized by cell number.

Creatine riboside levels were stratified as high and low by the median for breast cancer samples, lung tissue samples and pancreatic cancer samples, or by the 75th percentile of population control (i.e. cancer-free individuals) levels for urine

samples for lung and liver cancer cases. For breast and pancreatic cancer samples, which contained only samples from patients with cancer, the creatine riboside levels were stratified by the median. For cell lines, classification of creatine riboside levels as high or low was defined using the median value of the panel of cell lines tested. To test the association of driver mutations with creatine riboside levels in cell lines, mutation data were obtained for each cell line from the Cancer Cell Line Encyclopaedia and a chi square test was performed to test for the association of creatine riboside with driver mutations.

Gas Chromatography Coupled Mass Spectrometry Detection of Ribose

Quantitative detection of ribose, glucose and their heavy-labeled forms was performed using gas chromatography coupled mass spectrometry (GC-MS).

For this analysis, A549 and H460 cell extracts were prepared as follows. Cell pellets were resuspended in 0.3 ml 70% acetonitrile:water (70:30 v/v) containing 20 µl of DL-norleucine (Internal standard,10µM), followed by homogenization using a Precellys homogenizer (Bertin Instruments, Montigny-le-Bretonneux, France), utilizing 1.0 mm zirconia/silica beads for 30 sec at 6500 rpm. The samples were then centrifuged at 20,000g for 15 min at 4˚C and 250 µl of supernatant was taken and dried in a SpeedVac concentrator at room temperature. Cell media samples were prepared as follows: To 0.15 ml of conditioned cell culture media, 0.15 ml of acetonitrile containing with internal standard DL-norleucine (10µM) was added. The samples were centrifuged at 20,000g for 10 min at 4°C and 0.250 mL supernatant transferred to 2 ml vials. The dried residue from cell pellets or media samples were derivatized by adding

50 µl BSTFA and sonicated for 30 min at room temperature. The samples were diluted with 50 µl acetonitrile, briefly vortexed for 10 s and 1.00 µl was injected into the GC-MS using an autosampler.

D-ribose, U-$^{13}C_5$ D-ribose, D-glucose, and U-$^{13}C_6$ D-glucose chromatography were carried out on a capillary column (30 m × 0.250 mm, 0.25 µm; Agilent Technologies, Foster City, CA) with 17.0 min run time. All analytes were separated at 9.0 min retention time, m/z 217 (qualifier ions m/z 204, 191) TMS derivatives for D-ribose, at 9.0 min retention time, m/z 220 (qualifier ions m/z 206, 192) TMS derivatives for U-$^{13}C_5$ D-ribose, at 10.7 min retention time, m/z 204 (qualifier ions m/z 217, 191) TMS derivatives for D-glucose, at 10.7 min retention time, m/z 206 (qualifier ions m/z 220, 192) TMS derivatives for U13C6 D-Glucose and at 11.4 min retention time, m/z 200 (qualifier ions m/z 274, 302) TMS derivatives for internal standard DL-Norleucine on SIM mode of 40–500 m/z. Analyses were performed with an Agilent 6890N gas chromatograph coupled to an Agilent 5973 mass-selective detector (MSD) with the following chromatographic conditions: Initial temperature 100˚C for 2 min, increasing to 200˚C at 10˚C/min for 17 min. The front inlet temperature was 230˚C operating with a split ratio of 1:25. MSD ion source and interface temperature were 230˚C. The MSD operated in EI mode at 70 eV. Carrier gas was He (1.0 ml/min). GCMS data were acquired and processed using Agilent MassHunter WorkStation software. Standard solutions were used for precise quantitative determination of metabolite concentration. Stock solutions of calibration standards and internal standards (D-ribose, U-$^{13}C_5$ D-ribose, Dglucose, and U-$^{13}C_6$ D-glucose, DL-norleucine (5.0 mM in water) were prepared in

acetonitrile:water (50:50 v/v) for a final standard curve concentration range of 0.02-10μM, and internal standard concentration of 10μM.

RNA Sequencing

Lung tumor and adjacent non-tumor tissue samples and urine samples were collected as part of the NCI- University of Maryland Study according to protocols approved by the institutional review board. Total RNA was extracted from the tissues as previously described (2), and libraries suitable for paired-ended sequencing were generated using Illumina's TruSeq Stranded Total RNA Library Prep Kit. RNA samples were pooled and sequenced on a HiSeq. The sequencing quality of each sample was assessed using FastQC (3). Read- and alignment-level quality were assessed using FastQ Screen (4), Preseq (5) Picard tools, RSeQC (6) and QualiMap (7). Reads were trimmed for adapter sequences and low-quality bases using Cutadapt (8) prior to alignment against the human reference genome, hg19, using STAR (9) in two-pass mode with GENCODE v19 annotation (10) Gene expression levels were quantified using RSEM (11) Batch correction was performed using the Combat algorithm from the SVA package (12).

For analysis of TCGA data, RNAseq and clinicodemographic data of lung adenocarcinoma, lung squamous non-small cell lung cancer and hepatocellular carcinoma were downloaded from https://gdc.cancer.gov/about-data/publications/pancanatlas using survival information from (13). Corresponding mutational information was downloaded from GDAC FireHose (14–16). Tumors were assigned as Creatine Riboside High or Low depending on their transcriptional profile for the CR signatures identified in the NCI-MD

cohorts. Pentose Phosphate Pathway activity score was assigned to each sample using GSVA and the signature of G6PD, TALDO1, PRPS1 and RPE). For lung tumors, a mitochondrial urea cycle dysfunction score was assigned as CPS1 + NAGS expression. For hepatocellular carcinoma, the mitochondrial urea cycle activity score was assigned using GSVA for the mitochondrial urea cycle signature of CPS1, OTC and NAGS. Tumors were tertile stratified for each measure as low, medium and high. To ensure consistency with the CR signatures identified in CR-High and CR-Low tumors of the NCI-MD lung and liver cohorts, tumors assigned as CR-Low-Like tumors were high in the mitochondrial urea cycle score and low in the pentose phosphate pathway score. Tumors assigned as CR-High-Like tumors were low in the mitochondrial urea cycle score and high in the pentose phosphate pathway score. This stratification was carried through subsequent analyses in the same manner as the NCI-MD cohort analyses, as described in detail below.

Association with survival was assessed by log-rank test as well as cox proportional hazards models implemented using the survival and survminer packages in R. Differential gene expression analysis was performed using EdgeR (17) and Limma (18). Enriched pathways were identified using Gene Set Enrichment Analysis (GSEA) against the KEGG subset of the C2 MSigDb collection (19). Cell type deconvolution was performed using Cibersortx and the LM22 gene signature set of hematopoietic cell subsets in bulk tissues (20, 21).

Whole Exome Sequencing

Total DNA was extracted from lung tumor tissue from the NCI-MD cohort as described previously (2). Samples were pooled and sequenced on NovaSeq using Illumina Agilent SureSelect Human All Exon V7 in paired end-sequencing mode. The samples were trimmed, mapped and variants were called in the samples using the Dragen pipeline. Raw sequencing depth coverage over the target region was between 622x and 1,629x and mapped sequencing depth coverage over target (after alignment and marking duplicates) was between 411x to 1,070x. More than 98% of the target region had coverage greater than 30x. The somatic variants were called using tumor only analysis with the panel of normal. SNPs and INDELS were called using the GATK haplotype caller which is masked for off-target calls and filtered using QD>2.0, FS>60 for SNPs and QD<2.0, FS>200 for INDELS.

Stable Isotope Tracing

Metabolic pathway activity in creatine riboside low and -high cancer cells was examined by tracing stable heavy carbons from glucose through intracellular metabolite pools. Cells were cultured in phenol-red-free RPMI media containing 10% dialyzed serum during labeling experiments. Media was dialyzed as previously described (22). Cells were washed quickly using 0.9% NaCl and harvested in 80% aqueous methanol containing 500nM 15N, 13C- amino acid mix (Cambridge Isotopes Laboratories MSK-A2-1.2). Samples were dried (speedvac) before being resuspended in 100 ul LC/MS grade water and

analyzed by LC/MS as described (23). Metabolite profiling and stable isotope tracing LC/MS analyses were conducted on a QExactive bench top orbitrap mass spectrometer equipped with an Ion Max source and a HESI II probe, which was coupled to a Dionex UltiMate 3000 HPLC system (Thermo Fisher Scientific, San Jose, CA). External mass calibration was performed using the standard calibration mixture every 7 days. Dried polar metabolite extracts were reconstituted in 100 uL water and 2 uL were injected onto a SeQuant® ZIC®-pHILIC 150 x 2.1 mm analytical column equipped with a 2.1 x 20 mm guard column (both 5 mm particle size; EMD Millipore). Buffer A was 20 mM ammonium carbonate, 0.1% ammonium hydroxide; Buffer B was acetonitrile. The column oven and autosampler tray were held at 25°C and 4°C, respectively. The chromatographic gradient was run at a flow rate of 0.150 mL/min as follows: 0-20 min: linear gradient from 80-20% B; 20-20.5 min: linear gradient form 20-80% B; 20.5-28 min: hold at 80% B. The mass spectrometer was operated in full-scan, polarity-switching mode, with the spray voltage set to 3.0 kV, the heated capillary held at 275◦C, and the HESI probe held at 350◦C. The sheath gas flow was set to 40 units, the auxiliary gas flow was set to 15 units, and the sweep gas flow was set to 1 unit. MS data acquisition was performed in a range of *m/z* = 50-750, with the resolution set at 70,000, the AGC target at $1x10^6$, and the maximum injection time at 20 msec. Relative quantitation of polar metabolites was performed with XCalibur QuanBrowser 2.2 (Thermo Fisher Scientific) using a 5 ppm mass tolerance and referencing an in-house library of chemical standards. For stable isotope tracing analyses, data were corrected for natural abundance using an in-house script, as discussed in Buescher et al. (2015).

Tissue Preparation for Imaging Mass Spectrometry

Tumor and adjacent non-tumor tissue samples were prepared for *in situ* spatial measurement of creatine riboside levels using imaging mass spectrometry. Small blocks of fresh frozen human lung tumor tissues were shipped on dry ice and kept at -80°C prior to use. Tumor/non-tumor tissue pairs from each patient were analyzed. Tissues were placed in a -20°C cryostat (CM 1900, Leica Biosystems, Buffalo Grove, IL) to equilibrate prior to sectioning. Tissues were cut on the cryostat into 12 µm thick sections for imaging mass spectrometry and H&E staining. H&E stained sections were blinded and annotated as tumor and non-tumor regions by a pathologist (J. Beck) for cross-reference to the imaging mass spectrometry signal. Additionally, up to twelve additional sections were obtained at 10 µm for subsequent specialized staining and immunohistochemistry. The tissue sections designated for imaging mass spectrometry were thaw-mounted onto indium-tin oxide coated glass slides. The tissue sections were left to dry for at least 15 min in a desktop vacuum desiccator prior to matrix application. 2,5Dihydroxybenzoic acid (DHB, 40 mg/ml, 70% methanol with 0.1% trifluoroacetic acid) was used as the matrix and was applied to the tissue sections using an automated sprayer (TM Sprayer, HTX Technologies, Chapel Hill, NC). Eight passes were applied at 85°C in a criss-cross pattern, with a 1300 mm/min stage velocity, 2 mm track spacing and 0.15 ml/min flow rate.

Imaging Mass Spectrometry

The in situ spatial distribution of creatine riboside levels within tumor and adjacent non-tumor tissue was measured by imaging mass spectrometry. Tissue samples prepared as described above were analyzed in positive ion mode with a linear ion trap mass spectrometer equipped with a MALDI source and a nitrogen laser (LTQ XL, Thermo Scientific, Waltham, MA). A targeted MS/MS method was optimized for creatine riboside using a standard compound. Pseudo-selected reaction monitoring mode was used for imaging by isolating the precursor ion at m/z 264 with a 1 Da window, fragmenting it, and acquiring a full product ion mass spectrum at each pixel. Reconstructed ion images were generated in ImageQuest software (Thermo Scientific, Waltham, MA) by plotting the intensity of the main diagnostic fragment ion at m/z 132 as a function of location across the tissue surface. After imaging, the matrix was removed and the tissue section that had been imaged was stained with hematoxylin and eosin (H&E) for better image registration with tissue morphology. Images were acquired at 70 µm spatial resolution by using a pinwheel filter in front of the laser beam to reduce its diameter.

OPAL Multiplex Immunofluorescence Staining and Imaging

Slides were removed from -80C, brought to room temperature for 30 minutes, and fixed in a formaldehyde glutaraldehyde fixative for 30 minutes. After rinsing in T-TBS buffer, antigen retrieval was performed with Target Retrieval Solution (Agilent #S2367) at 100C for 15 minutes in a Biocare Decloaking Chamber. Slides were cooled for 15 minutes, rinsed with distilled water and placed in T-TBS buffer. Slides for autofluorescence controls were set aside during the following

antibody stains but were subjected to each round of heat treatment. Sections for multiplex staining were circled with pap pen, blocked with 2.5% normal horse serum (NHS) for 20 minutes, followed by incubation with Ki67 (Cell Signaling Technology #9027, diluted 1:25) for 30 minutes. Slides were rinsed in T-TBS, incubated with the ImmPRESS a/Rabbit-HRP reagent, rinsed, followed by OPAL Polaris 480. Antibody/HRP stripping was performed again as described above. The next round of staining was performed with NHS block, PD-1 (Cell Signaling Technology #43248, diluted 1:100) overnight at 4C, ImmPRESS Reagent Horse anti-Mouse IgG-HRP (Vector Laboratories), and OPAL 570. Antibody/HRP stripping was again performed, followed by a subsequent round of staining with NHS block, CD68 (Agilent #M0876, diluted 1:50) for 30 minutes, ImmPRESS a/Mouse-HRP reagent, and OPAL 620. Following the antibody/HRP stripping, the final round of staining with NHS block, CD8 (abcam #ab101500, diluted 1:50) was performed for 30 minutes, ImmPRESS a/Rabbit-HRP reagent, OPAL TSADIG, antibody stripping, OPAL Polaris 780, and spectral DAPI. Multiplexstained and autofluorescence control slides were coverslipped with ProLong Gold Antifade Mountant (Invitrogen). Slides were kept flat in a slide folder overnight at room temperature then transferred to -20C until imaging. Slides were imaged on a Vectra Polaris. Multiplexed images were unmixed using InForm software. Quantitative analysis of cell types was performed in QuPath (v2.4). Tissue masks were generated by pixel detection. Cell detection was performed on the DAPI channel using StarDist. Feature detection algorithms were manually trained on composite images from 6-7 tissues and applied equally to all images. Regions of sections that were folded were excluded from the final analysis. Pathological annotations performed on the MALDI imaging mass

spectrometry sections were applied to the multispectral images to correlate creatine riboside signal with immunofluorescence markers. The number of positive cells were normalized to the total number of cell detections in that region of interest.

Supplemental Figures and Figure Legends

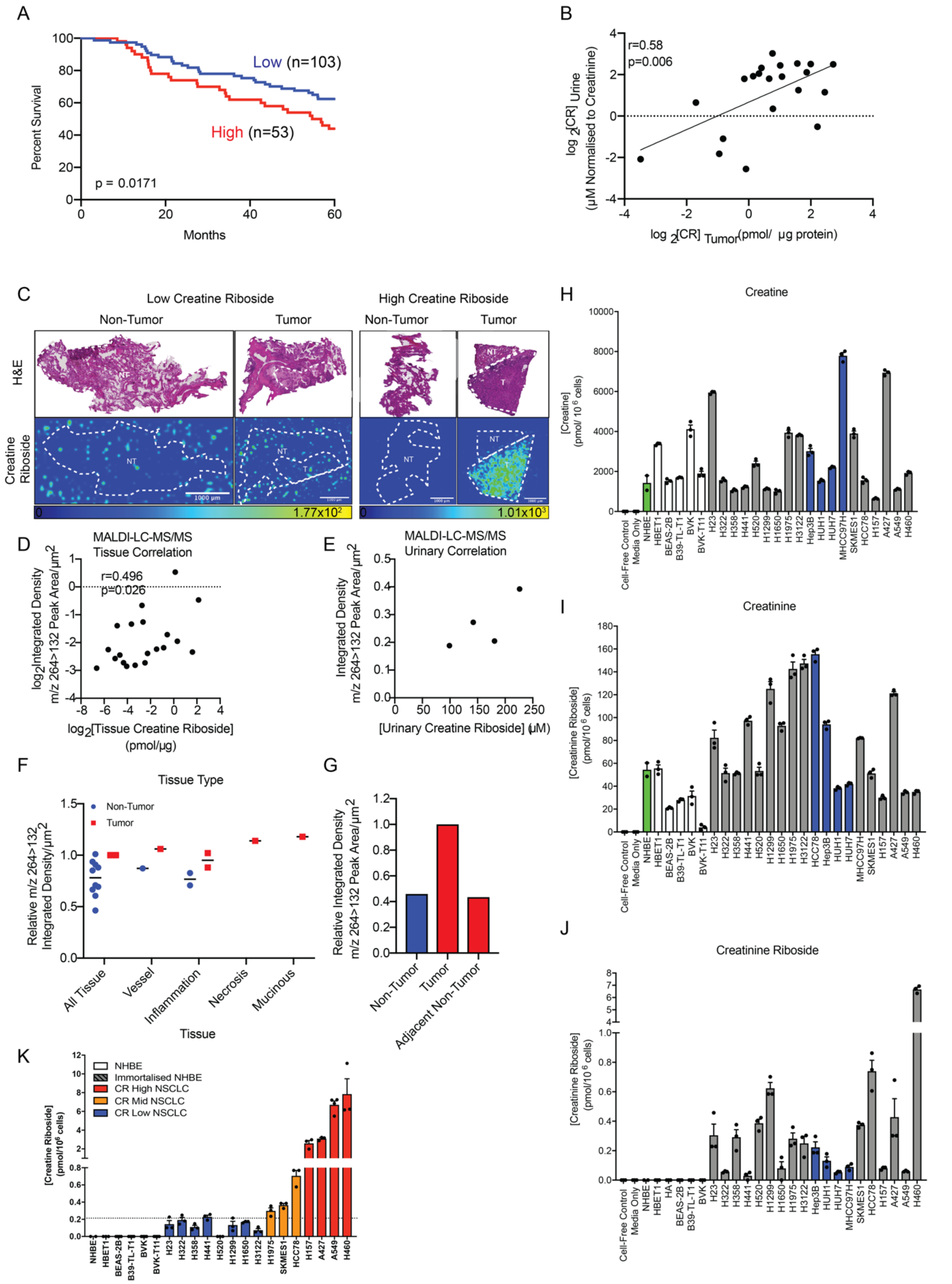

Supplemental Figure 1. A) Survival analysis of urinary CR levels in NSCLC patients as measured using the highly quantitative ^{13}C-CR assay (CR Low = lowest 66%ile

(n=103); CR High = highest 33%ile (n=53); log rank test p=0.0171). **B)** Correlation of urinary CR with tumor tissue CR levels. Spearman's correlation R=0.6, p=0.006, n=21 NSCLC tumor tissues. **C)** MALDI MS image of tumor and non-tumor tissue presenting the same data as Figure 1C but with the CR signal of each tumor and non-tumor pair presented on individual scales to illustrate the range within individual patients. **D and E)** Correlation of the MALDI-IMS CR signal abundance with the tissue CR concentration (Spearman's correlation r=0.496, p = 0.026) **(D)** and the urinary CR concentration (Spearman's correlation r=0.773) **(E)** as measured by LCMS. **F)** CR abundance within different regions of the tissues as measured by MALDI-imaging mass spectrometry. Blue: non-tumor; Red: tumor. **G)** CR abundance in a tumor tissue section that contains non-tumor tissue surrounded by non-tumor tissue ("Adjacent Non-tumor") compared with noninvolved non-tumor tissue ("Non-tumor"). **H, I and J)** Intracellular concentrations of creatine **(H)**, creatinine **(I)** and creatinine riboside **(J)** in the panel of cell lines tested. NHBE: normal bronchial epithelial cells (green); immortalized NHBE: immortalized normal human bronchial epithelial cells (white); NSCLC: NSCLC cells (grey); HCC: hepatocellular carcinoma (blue). Mean ± SEM of n=3-4 independent experiments. Metabolite concentrations were measured by LC-MS/MS and normalized to cell number. **K)** Stratification of NSCLC cell lines as low, mid and high endogenous CR levels. Dotted line indicates the median creatine riboside concentration. Mean ± SEM of n=3-4 independent experiments.

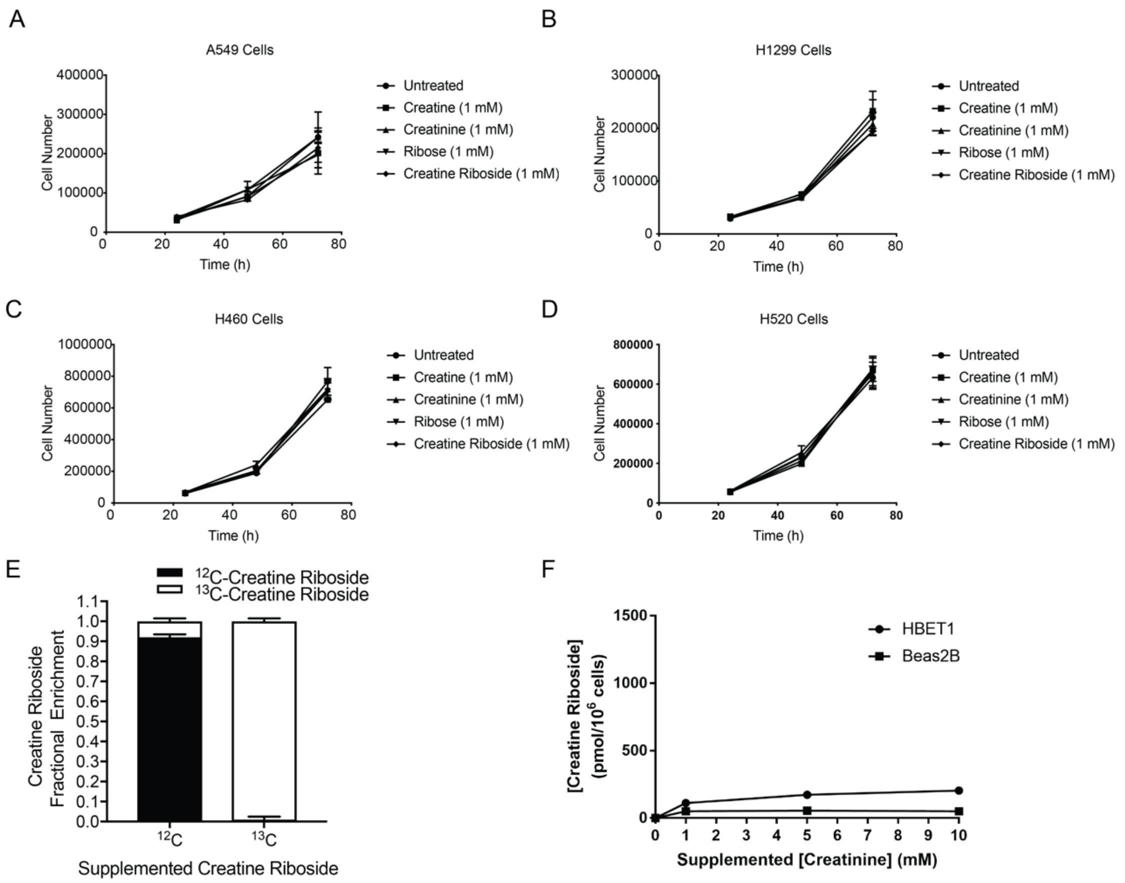

Supplemental Figure 2. **A-D)** Proliferation rate of CR^{High} (A549 **(A)**; H460 **(C)**) and CR^{Low} (H1299 **(B)**; H520 **(D)**) cell lines when cultured in normal media (untreated) or normal media supplemented with 1mM creatine, creatinine, ribose or CR. 3 independent experiments. No significant difference in proliferation. **E)** Fractional enrichment of the intracellular pool of CR in H460 cells cultured in media supplemented with ^{12}C-CR or ^{13}C-CR. **F)** CR levels in immortalized bronchial epithelial cells (HBET1 (circles) and Beas2B (squares)) supplemented with exogenous creatinine.

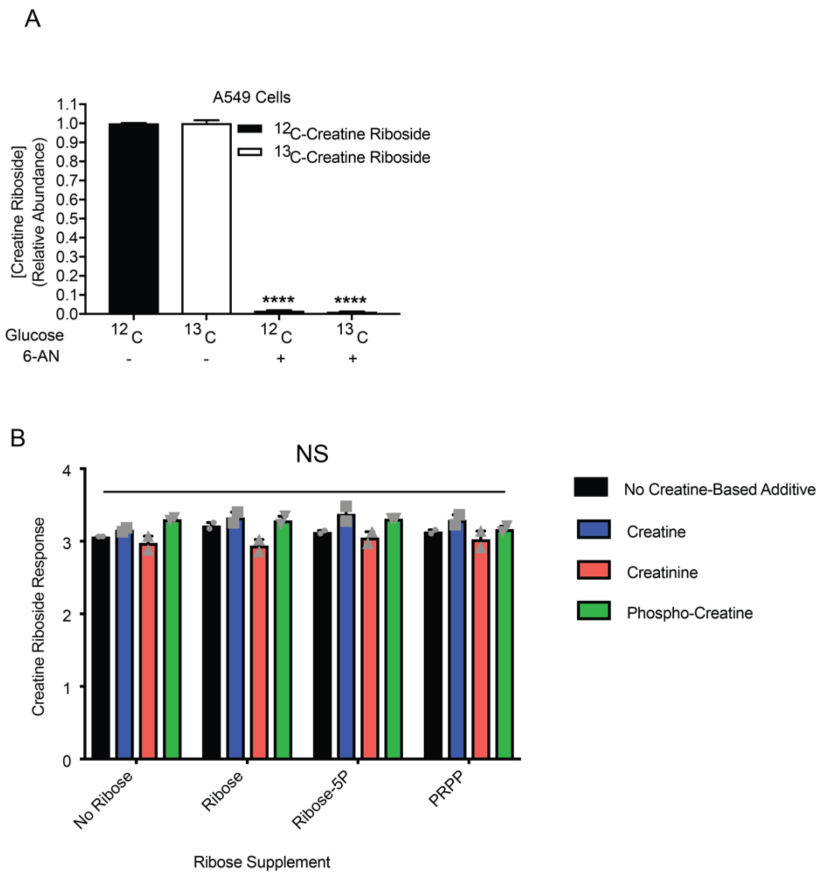

Supplemental Figure 3. A) Fractional enrichment of CR labeling from ^{13}C-Glucose over time in H460 cells treated with (black line) and without (blue line) the PGDH inhibitor 6-aminonicotinamide (6-AN). Mean±SEM of n=3 independent experiments, *p<0.05, Two-way ANOVA with Holm-Sidak multiple comparison correction. **B)** Relative CR concentrations in urine samples spiked with ribose metabolites and creatine metabolites prior to extraction. Mean ± SEM of n=2 independent experiments, Two-way ANOVA with Holm-Sidak multiple comparison correction, no significant difference compared with unsupplemented urine.

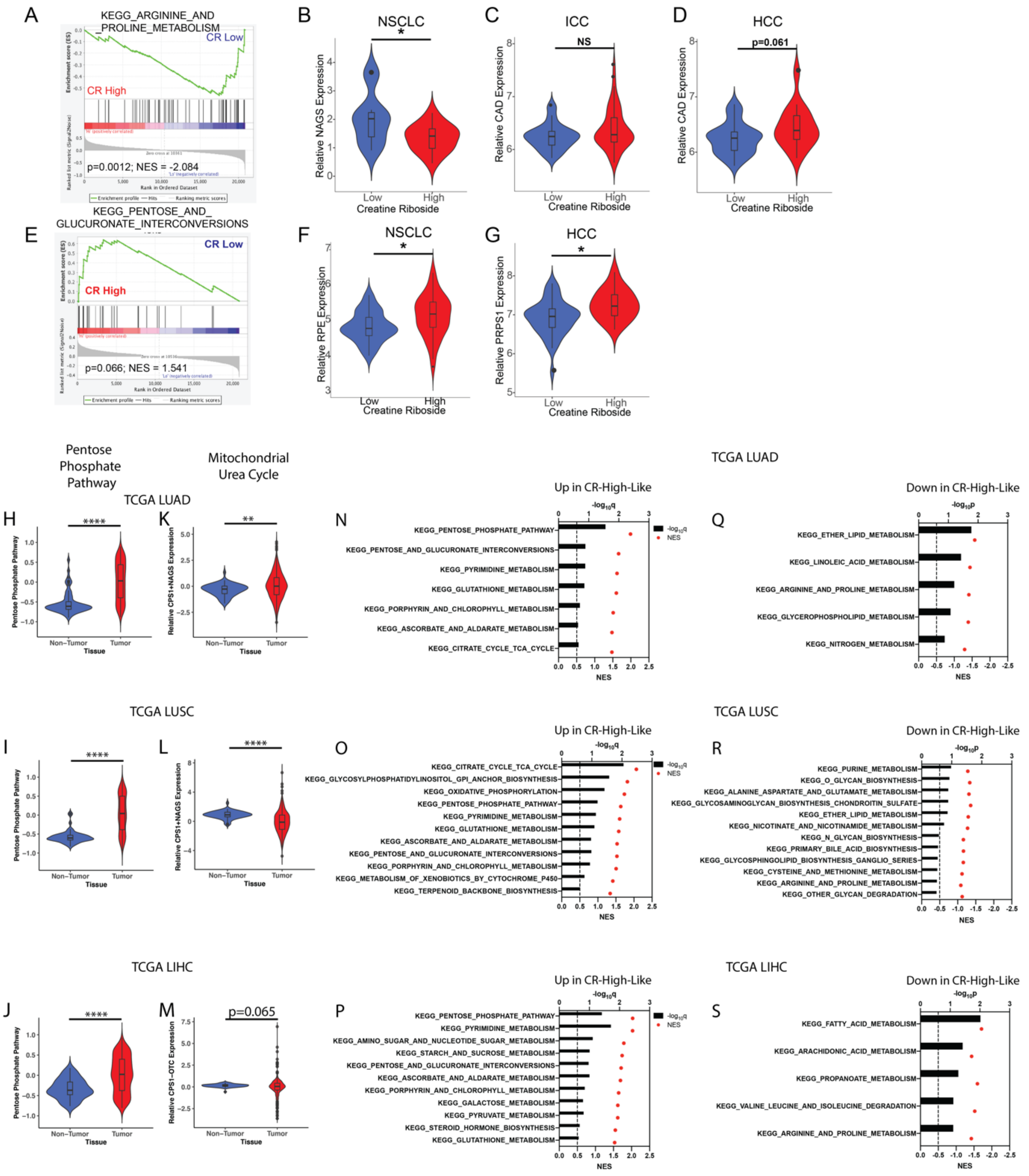

Supplemental Figure 4. A-D) Gene Set Enrichment Plot of arginine and proline metabolism in NSCLC **(A)** with expression of core enriched genes in NSCLC **(B),** intrahepatic cholangiocarcinoma **(C)** and hepatocellular carcinoma **(D)**. **E-G)** Gene Set Enrichment Plot of the pentose and glucuronate interconversion pathway in NSCLC **(E)** and expression of core enriched genes in NSCLC **(F)** and hepatocellular carcinoma **(G)**. **H-J)** Gene Set variation Analysis of pentose phosphate pathway

expression in the TCGA cohorts of lung adenocarcinoma **(H)** , squamous non-small cell lung cancer **(I)** and hepatocellular carcinoma **(J)** in tumor compared with non-tumor tissue. ****p<0.001, Mann-Whitney U-test. **K-M)** Mitochondrial urea cycle dysfunction in the TCGA cohorts of lung adenocarcinoma **(K;** as measured by CPS1+NAGS expression), squamous non-small cell lung cancer **(L;** as measured by CPS1+NAGS expression) and hepatocellular carcinoma **(M;** as measured by CPS1-OTC expression**)** in tumor compared with non-tumor tissue. ** p<0.01, ****p<0.001, Mann-Whitney U-test. **N-P)** Gene Set Variation Analysis of top KEGG metabolic pathways enriched in CR^{High}-Like tumors in the TCGA cohorts of lung adenocarcinoma **(N)**, squamous non-small cell lung cancer **(O)** and hepatocellular carcinoma **(P).** Black bars: -$\log_{10}$(P-values adjusted for multiple comparisons); red dots: normalized enrichment score. **Q-S)** Gene Set Enrichment Analysis results of the top KEGG metabolic pathways enriched in CR^{High}-Like tumors in the TCGA cohorts of lung adenocarcinoma **(Q)**, squamous non-small cell lung cancer **(R)** and hepatocellular carcinoma **(S).** Black bars: -$\log_{10}$(P-values); red dots: normalized enrichment score.

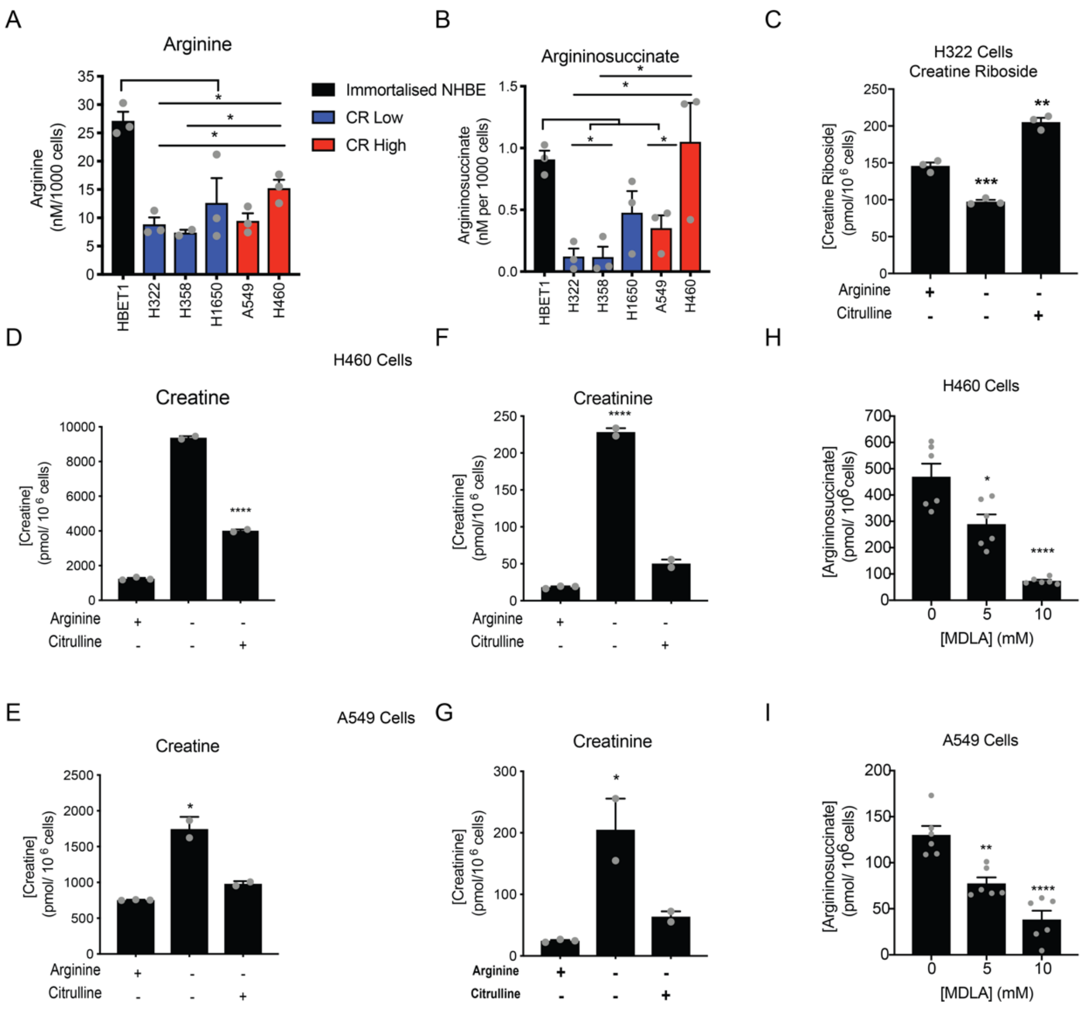

Supplemental Figure 5. A and B) Intracellular arginine **(A)** and argininosuccinate **(B)** concentrations in CR^{High} (red) and CR^{Low} (blue) NSCLC cell lines and immortalized bronchial epithelial cells (black). Mean ± SEM of n=3 independent experiments, *p<0.05, One-way ANOVA with Dunnett's multiple comparison correction. **C)** Intracellular CR levels in the CR^{Low} (H322) NSCLC cell line cultured in creatinine-enriched normal or arginine-depleted media with or without citrulline supplementation. Mean ± SEM of n=3 independent experiments, *p<0.05, One-way ANOVA with Dunnett's multiple comparison correction. **D-G)** Intracellular creatine **(D, E)** and creatinine **(F, G)** concentrations of H460 **(D, F)** and A549 **(E, G)** cells in response to arginine deprivation and supplementation with citrulline. Mean ± SEM of n=2 independent experiments, *p<0.05, ****p<0.0001, One-way ANOVA with

Dunnett's multiple comparison correction. **H and I)** Intracellular argininosuccinate concentrations in H460 **(H)** and A549 **(I)** cells treated with methyl –DL-aspartate (MDLA). Mean ± SEM of n=2 independent experiments, *p<0.05, **p<0.01, ****p<0.0001 relative to untreated cells, One-way ANOVA with Dunnett's multiple comparison correction.

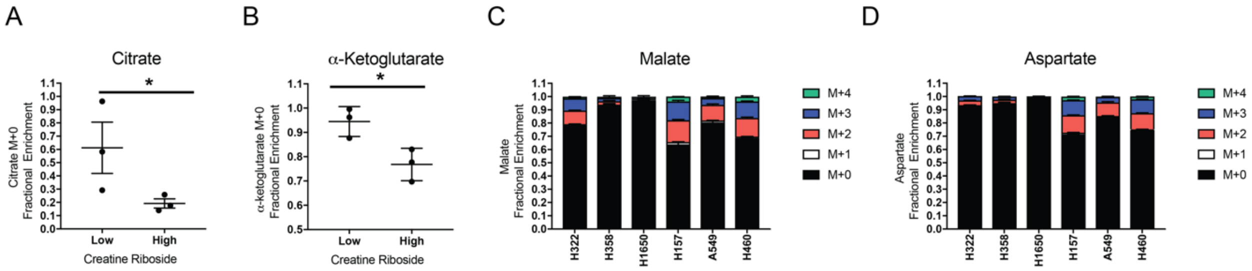

Supplemental Figure 6. A and B) Fractional enrichment of unlabeled tricarboxylic acid metabolites citrate **(A)** and alpha-ketoglutarate **(B)** in NSCLC cell lines with CR^{Low} and CR^{High} levels incubated with U-^{13}C-glucose. Mann Whitney Utest *$p<0.05$. **C and D)** Labeling pattern of unlabeled tricarboxylic acid metabolites malate **(C)** and aspartate **(D)** in CR^{Low} and CR^{High} NSCLC cell lines incubated with U-^{13}C-glucose.

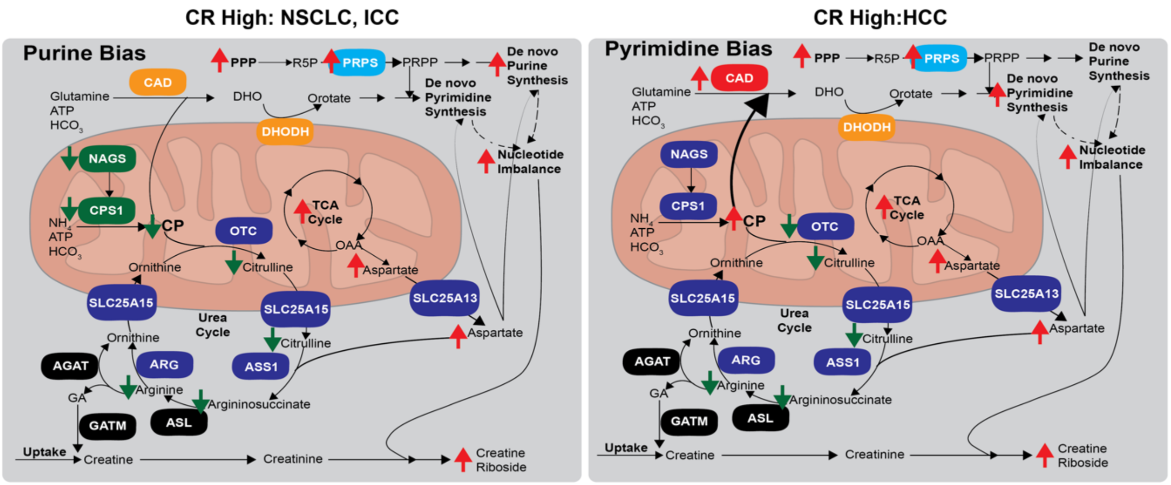

Supplemental Figure 7. Schematic of the metabolic phenotypes promoting CR production in tumors with a purine bias (NSCLC, ICC; left panel) and pyrimidine bias (HCC; right panel).

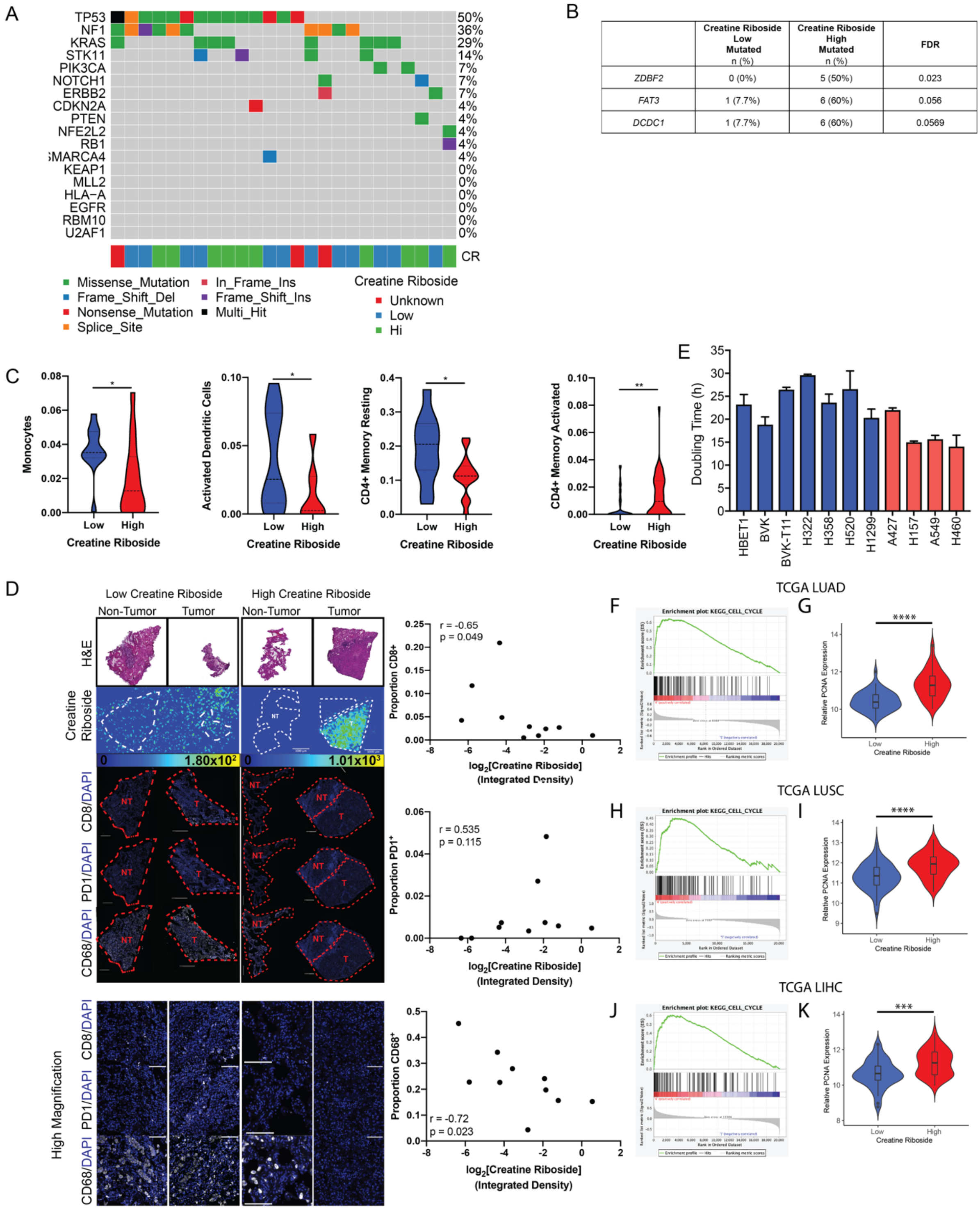

	Creatine Riboside Low Mutated n (%)	Creatine Riboside High Mutated n (%)	FDR
ZDBF2	0 (0%)	5 (50%)	0.023
FAT3	1 (7.7%)	6 (60%)	0.056
DCDC1	1 (7.7%)	6 (60%)	0.0569

Supplemental Figure 8. A) Mutational frequencies in the NCI-MD NSCLC cohort according to CR levels (lower panel). **B)** Top most differentially enriched mutant genes in CR^{High} vs CR^{Low} NSCLC tumors. **C)** Immunological

differences in CR^{High} NSCLC tumors compared with CR^{Low} NSCLC tumors, as measured by bulk RNAseq deconvolution (Mann-Whitney U-test, *p<0.05). **D)** Immunological differences in CR^{High} liver tumors compared with CR^{Low} lung tumors, as measured by multiplex immunohistochemistry, in tumor and nontumor samples for which creatine riboside levels were spatially characterized by imaging mass spectrometry (Figure 1C) (MALDI imaging mass spectrometry creatine riboside quantitation presented in upper panels). Correlation of creatine riboside level with number of CD8+, PD1+ and CD68+ cells in tumor samples only (right panel) show significant negative correlation of CD8+ (r=-0.65, p=0.049) and CD68+ cells (r=-0.72, p=0.023) with tumoral creatine riboside levels. The positive correlation between creatine riboside levels and PD1+ cells did not reach significance (r=0.535, p=0.115). Spearman's correlation. Images are representative of n=10 matched tissues (DAPI = blue, markers of interest overlaid in greyscale; Low magnification scale bar = 500 µm; High magnification scale bar = 100 µm). NT = non-tumor, T = tumor. **E)** Doubling time measurements for individual CR^{High} (red) and CR^{Low} (blue) NSCLC cell lines as measured by Trypan blue and cell counting. Corresponds to the group summary data presented in Figure 9D). Mean ± SEM of n=2 independent experiments. **F, H and J)** Gene set enrichment analysis results of CR^{High}-Like and CR^{Low}-Like tumors in the TCGA cohorts of lung adenocarcinoma (TCGA LUAD, **F**), squamous non-small cell lung cancer (TCGA LUSC, **H**) and hepatocellular carcinoma (TCGA LIHC, **J**) identified that CR^{High} tumors are significantly enriched in the expression of cell cycle genes compared with CR^{Low} tumors. **G, I and K)** Cell proliferation marker PCNA expression in CR^{High}-Like and CR^{Low}-Like tumors in the TCGA cohorts of lung adenocarcinoma (TCGA LUAD, **G**), squamous non-small cell lung cancer (TCGA LUSC, **I**) and

hepatocellular carcinoma (TCGA LIHC, **K**). *** $p<0.001$, ****$p<0.0001$, Mann-Whitney U-test.

References

1. Patel DP et al. Improved detection and precise relative quantification of the urinary cancer metabolite biomarkers - Creatine riboside, creatinine riboside, creatine and creatinine by UPLC-ESI-MS/MS: Application to the NCI-Maryland cohort population controls and lung cancer cases. *J. Pharm. Biomed. Anal.* 2020;191:113596.

2. Chaisaingmongkol J et al. Common molecular subtypes among asian hepatocellular carcinoma and cholangiocarcinoma. *Cancer Cell* 2017;32(1):57–70.e3.

3. Andrews S. FastQC: A Quality Control Tool for High Throughput Sequence Data [Internet]2010;http://www.bioinformatics.babraham.ac.uk/projects/fastqc/. cited June 1, 2018

4. Wingett SW, Andrews S. FastQ Screen: A tool for multi-genome mapping and quality control. *F1000Res.* 2018;7:1338.

5. Daley T, Smith AD. Predicting the molecular complexity of sequencing libraries. *Nat. Methods* 2013;10(4):325–327.

6. Wang L, Wang S, Li W. RSeQC: quality control of RNA-seq experiments. *Bioinformatics* 2012;28(16):2184–2185.

7. Okonechnikov K, Conesa A, García-Alcalde F. Qualimap 2: advanced multi-sample quality control for high-throughput sequencing data. *Bioinformatics* 2016;32(2):292–294.

8. Martin M. Cutadapt removes adapter sequences from high-throughput sequencing reads. *EMBnet j.* 2011;17(1):10.

9. Dobin A et al. STAR: ultrafast universal RNA-seq aligner.. *Bioinformatics* 2013;29(1):15–21.

10. Harrow J et al. GENCODE: the reference human genome annotation for The ENCODE Project. *Genome Res.* 2012;22(9):1760–1774.

11. Li B, Dewey CN. RSEM: accurate transcript quantification from RNA-Seq data with or without a reference genome. *BMC Bioinformatics* 2011;12:323.

12. Leek JT, Johnson WE, Parker HS, Jaffe AE, Storey JD. The sva package for removing batch effects and other unwanted variation in high-throughput experiments. *Bioinformatics* 2012;28(6):882–883.

13. Liu J et al. An Integrated TCGA Pan-Cancer Clinical Data Resource to Drive High-Quality Survival Outcome Analytics. *Cell* 2018;173(2):400–416.e11.

14. Cancer Genome Atlas Research Network. Comprehensive genomic characterization of squamous cell lung cancers. *Nature* 2012;489(7417):519–525.

15. Wheeler DA, Roberts LR, The Cancer Genome Atlas Research Network. Comprehensive and integrative genomic characterization of hepatocellular carcinoma. *Cell* 2017;169(7):1327–1341.e23.

16. Cancer Genome Atlas Research Network. Comprehensive molecular profiling of lung adenocarcinoma. *Nature* 2014;511(7511):543–550.

17. McCarthy DJ, Chen Y, Smyth GK. Differential expression analysis of multifactor RNA-Seq experiments with respect to biological variation. *Nucleic Acids Res.* 2012;40(10):4288–4297.

18. Ritchie ME et al. limma powers differential expression analyses for RNA-sequencing and microarray studies. *Nucleic Acids Res.* 2015;43(7):e47.

19. Subramanian A et al. Gene set enrichment analysis: a knowledge-based approach for interpreting genome-wide expression profiles. *Proc. Natl. Acad. Sci. USA*

2005;102(43):15545–15550.

20. Newman AM et al. Determining cell type abundance and expression from bulk tissues with digital cytometry. *Nat. Biotechnol.* 2019;37(7):773–782.

21. Newman AM et al. Robust enumeration of cell subsets from tissue expression profiles. *Nat. Methods* 2015;12(5):453–457.

22. Cantor JR et al. Physiologic medium rewires cellular metabolism and reveals uric acid as an endogenous inhibitor of UMP synthase. *Cell* 2017;169(2):258–272.e17.

23. Birsoy K et al. An essential role of the mitochondrial electron transport chain in cell proliferation is to enable aspartate synthesis. *Cell* 2015;162(3):540–551.
